# Entry of antiepileptic drugs (valproate and lamotrigine) into the developing rat brain

**DOI:** 10.12688/f1000research.52607.1

**Published:** 2021-05-13

**Authors:** Samuel J. Toll, Fiona Qiu, Yifan Huang, Mark D. Habgood, Katarzyna M. Dziegielewska, Shuai Nie, Norman R. Saunders

**Affiliations:** 1 Biochemistry & Pharmacology, University of Melbourne, Parkville, Victoria, 3010, Australia; 2 Melbourne Mass Spectrometry and Proteomics Facility, Bio21 Institute, University of Melbourne, Parkville, Victoria, 3010, Australia

**Keywords:** CSF, blood-brain barrier, choroid plexus, efflux mechanisms, epilepsy, fetus, neonate, placenta.

## Abstract

**Background: **Women with epilepsy face difficult choices whether to continue antiepileptic drug treatment during pregnancy, as uncontrolled seizures carry great risk to mother and fetus but continuing treatment may have adverse effects on baby’s development. This study aimed at evaluating antiepileptic drug entry into developing brain.

**Methods: **Anaesthetised pregnant, non-pregnant adult females, postnatal and fetal rats were injected intraperitoneally with different doses, single or in combinations, of valproate and lamotrigine, all within clinical range. Injectate included ^3^H-labelled drug. After 30min, CSF, blood and brain samples were obtained; radioactivity was measured using liquid scintillation counting. Some animals were also exposed to valproate in feed throughout pregnancy and into neonatal period. Drug levels were measured by liquid chromatography coupled to mass spectrometry (LC-MS). Results are given as CSF or tissue/plasma% as index of drug entry.

**Results: **Entry of valproate into brain and CSF was higher at E19 and P4 compared to adult but was not dose-dependent;  placental transfer increased significantly at highest dose of 100mg/Kg. Lamotrigine entry into the brain was dose dependent only at E19. Chronic valproate treatment, or combination of valproate and lamotrigine had little effect on either drug entry, except for reduced valproate brain entry in adult brain with chronic treatment. Placental transfer decreased significantly after chronic valproate treatment. LC-MS measurement of valproate in adults confirmed that rat plasma values were within the clinical range and CSF/plasma and brain/plasma ratios for LC-MS and ^3^H-valproate were similar.

**Conclusion: **Results suggest that entry of valproate may be higher in developing brain, the capacity of barrier mechanism is mostly unaffected by doses within the clinical range, with or without addition of lamotrigine. Chronic valproate exposure may result in upregulation in cellular mechanisms restricting its entry into the brain. Entry of lamotrigine was little different at different ages and was not dose dependent.

## Abbreviations

ABC, ATP-binding cassette; CNS, central nervous system; CSF, cerebrospinal fluid; DPM, disintegrations per minute; E, embryonic (note that by longstanding convention all gestational ages in rodents are referred to as embryonic, but in this study E19 is a fetal stage); i.p., intraperitoneal; i.v., intravenous; LTG, lamotrigine; LC-MS, liquid chromatography coupled to mass spectrometry; P, postnatal; SD, standard deviation; μCi, micro Curie; VPA valproic acid.

## Introduction

Epilepsy is a neurological disorder characterised by seizures of varying types and severity ranging from absence, in which the patient becomes unresponsive to stimuli, to tonic–clonic or atonic seizures, where the individual suffers a loss of motor control ( Thijs *et al.*, 2019). Epilepsy affects people of all ages and sexes and it has been estimated that over 70 million people suffer from this disorder worldwide ( Thijs *et al*., 2019).

In some cases of focal epilepsy, surgical removal of the provoking brain region can provide a cure ( Rugg-Gunn *et al*., 2020; Thijs *et al*., 2019) but for many people epilepsy is a continuing condition requiring lifelong drug treatment. A particular clinical problem is that during pregnancy epileptic women need to continue their medication, otherwise the recurrence of seizures may adversely affect both their health and the health of their offspring ( Tomson *et al*., 2019). Much is known about adverse effects of antiepileptic drugs, particularly in terms of the occurrence of congenital malformations when these drugs are taken early in pregnancy; less is known about the longer-term effects on brain development and behaviour in the offspring. Because of a lack of a regulatory framework drugs used in pregnancy are prescribed “off-label”. When advising patients clinicians therefore have to rely on experience and limited clinical and animal experimental reports aggregated into databases ( *e.g.* Briggs *et al*., 2017). However, there is a general requirement ( *e.g.* by the US Federal Drug Administration) that all new drugs before being used in any patients should be tested in animals (usually rodents) for possible teratogenic effects. Such tests have shown that a well-established antiepileptic drug, valproate causes a significant number of congenital abnormalities in animals ( Jazayeri *et al*., 2020) but also in humans; Tomson *et al.* (2018) found that the risk for congenital malformation in children of mothers exposed to valproate was increased by 4–5-times especially at higher valproate doses, and particularly when used in the first trimester (see also Abou-Khalil, 2019; Vajda, 2012). The most common problems associated with gestational valproate use are cardiac malformations, hypospadias, renal defects, and neural tube defects, with higher doses increasing the risk of spina bifida ( Jentink *et al*., 2010). Possible ill-effects of taking the drug later in pregnancy have been little studied but will be outlined later in the Discussion. Because of the high incidence of congenital malformations occurring in offspring of pregnant women taking valproate, some countries ( *e.g.* France) have banned its use in pregnancy ( Casassus, 2017). Other advisory bodies urge caution and advise avoiding or limiting the use of valproate in pregnancy ( Australian Medicines Handbook, 2019; Briggs *et al*., 2017; The Royal Women’s Hospital Pregnancy and Breastfeeding Medicines Guide, 2020). The problem for clinicians and their patients is that valproate remains the most effective and in some cases, the only form of effective treatment for some forms of epilepsy ( Tomson *et al*., 2015). Currently clinicians deal with this problem by using the lowest doses of valproate that will still suppress seizures. This is often achieved by combining a lower dose of valproate with a second antiepileptic drug, for example lamotrigine ( Tomson *et al*., 2019). However, little is known about how these drugs interact in terms of entry into a fetus and its brain and still less about possible deleterious effects in the offspring. This paper describes an experimental approach using an established animal model (rat, Koehn *et al*., 2019; Koehn *et al*., 2020) aimed at shedding light on these problems. The starting point for such studies, as described here, is to determine the extent of age-related entry into the brain and CSF of valproate and lamotrigine used as mono- or combination therapies at doses which are within the clinical range.

## Methods

### Ethical statement

The Sprague–Dawley (RRID: RGD_728193) strain of *Rattus norvegicus* was used in this study. All animal experimentation was approved by the University of Melbourne Animal Ethics Committee (Ethics Permission AEC: 1714344.1) and conducted in compliance with Australian National Health and Medical Research Guidelines. All animals were assessed as healthy prior to commencement of experiments. All experiments were short term and conducted under anaesthesia. All efforts were made to ameliorate any suffering of animals. They were handled by experienced researchers in such a way as to minimise stress prior to being anaesthetised.

### Animals

Sprague–Dawley rats were supplied by the Animal Resources Centre, WA, and the University of Melbourne Biological Research Facility. Animals were kept in a 12-hour light/dark cycle. They were provided with *ad libitum* access to food, consisting of dry pellets of a fixed formulation for rats (supplied by Specialty Feeds, Western Australia), with the exception of animals designated for chronic valproate experiments (see below). All animals had access to a continuous supply of water. Animals were housed in groups of 2–4 (adults) per cage (25cm×35cm×25cm on Breeders Choice paper bedding, made from 99% recycled paper; it is biodegradable with no added chemicals).

Three ages were studied: fetuses from time-mated females (all primigravida) at embryonic day (E) 19, pups at postnatal day (P) 4, and adults. E19 is a stage of development when adequate volumes of blood and cerebrospinal fluid (CSF) can be obtained for analysis from fetal rats without pooling ( Dziegielewska *et al*., 1981); also individual pups can be injected intraperitoneally while still inside the uterine horn and kept viable for periods of time ( Koehn *et al*., 2019; Koehn *et al*., 2020). These ages represent developmental milestones that can be translated to stages of human brain development corresponding to the end of first trimester, late second trimester and adult ( Clancy *et al*., 2013; Workman *et al*., 2013). They can also be compared with data from previous studies of other drugs carried out at these ages ( Koehn *et al*., 2019; Koehn *et al*., 2020). All animals were injected with a drug dose of one of the following treatments: valproate, lamotrigine, or valproate and lamotrigine combined, with tracer amounts of ^3^H-labelled drug incorporated into the injectate (for details see Table 1). ^3^H-dextran was used to estimate residual blood amounts in brain tissue as described below. Animal numbers ( Table 2) were based on previous experience of such experiments and were the minimum number required to detect a significant difference between groups at p<0.05. Animals were selected for treatment groups to ensure weights were statistically similar between direct comparisons. Where possible, equal numbers of male and female fetuses and postnatal animals were used. Animals were allocated to experiments by the Animal House staff, who had no knowledge of the particular experiments to be performed. The experimenters had no role in the selection of the animals, thus avoiding selection bias. Most experiments were carried out between 09.00 and 14.00 hours. Very few experiments were unsuccessful; details are given in the legend to Table 2.

**Table 1.** Materials, sources and amounts used.

Material	Source	Amount			
		E19 Mother	E19 Fetus	P4	Adult
**Radiolabelled drugs** **& markers**					
^3^H-dextran	American Radiolabelled Chemicals Inc	-	0.5 µCi	1 µCi	6 µCi
^3^H-valproate	Moravek Inc	20–40 µCi	0.25 µCi	1–2 µCi	10–20 µCi
^3^H-lamotrigine	Moravek Inc	40 µCi	0.5 µCi	2 µCi	20 µCi
**Drugs & chemicals**					
Sodium valproate	Sigma-Aldrich	10, 30, 100 or 200 mg/kg body weight			
Lamotrigine	Sigma-Aldrich	6, 20 and 40 mg/kg body weight			
Isotonic NaCl solution	Pfizer				
Ethyl alcohol					
Heparin	Hospira Inc	5000 units per mL			
Isoflurane	Pharmachem	by inhalation			
Urethane	Sigma-Aldrich	25% *w*/ *v* 1.0 mL/100 g body weight			
Soluene 350	PerkinElmer	500 µL per sample			
Emulsifier Safe	PerkinElmer	5 mL per sample			
PVC tubing	Microtube Extrusions Pty Ltd				
Heparinised microhematocrit capillary	Bacto				
D6-valproate	Novachem	1.2 mg per sample			
HPLC grade methanol	Fisher chemical	80 µL per sample			

**Table 2.** Drugs, doses of lamotrigine (LTG) or valproate (VPA). Time between injection and collection, method of administration (MoA) and number of animals used at each age (n = number of litters); *i.p.* (intraperitoneal), *i.v.* (intravenous). Animal losses (not recorded here): One E19 mother from blood loss during cannulation. Three P4 animals not used (2 dose errors, one died before end of experiment). In the chronic valproate feed experiments approximately half of the fetuses died *in utero*.

Radio-labelled drug	Doses of cold drug (mg/Kg)		Time (min)	MoA	E19 n	P4 n	Adult n
	VPA	LTG					
^3^H-dextran	-	-	5	*i.v.*	6 (1)	5 (2)	3
^3^H-VPA	10	-	30	*i.p.*		5 (2)	4
^3^H-VPA	30	-	30	*i.p.*	To dam: 8 (1) To pup: 5 (1)	3 (1)	4
-	30	-	30	*i.p.*		4 (1)	3
^3^H-VPA	100	-	15	*i.p.*		2 (1)	
^3^H-VPA	100	-	30	*i.p.*	To dam: 6 (1) To pup: 5 (1)	3 (1)	3
-	100	-	30	*i.p.*		4 (1)	3
^3^H-VPA	100	-	60	*i.p.*		2 (1)	
^3^H-VPA	100	-	90	*i.p.*		2 (1)	
^3^H-VPA	200	-	30	*i.p.*		3 (1)	4
^3^H-LTG	-	6	30	*i.p.*	To dam: 12 (1)	3 (1)	3
^3^H-LTG	-	20	30	*i.p.*	To dam: 5 (1) To pup: 8 (2)	3 (2)	3
^3^H-LTG	-	20	60	*i.p.*		2 (1)	
^3^H-LTG	-	20	90	*i.p.*		2 (1)	
^3^H-LTG	-	20	120	*i.p.*		2 (2)	
^3^H-LTG	-	40	30	*i.p.*		3 (2)	
^3^H-VPA	30	2	30	*i.p.*		3 (1)	
^3^H-VPA	30	6	30	*i.p.*	To dam: 10 (1)	4 (2)	4
^3^H-VPA	30	12	30	*i.p.*		4 (1)	
^3^H-VPA	30	20	30	*i.p.*		4 (1)	
^3^H-LTG	30	6	30	*i.p.*		3 (1)	4
^3^H-VPA	Feed+ 20 g/kg	-	30	*i.p.*	To pup: 6 (1)	4 (1)	4

### Drugs and markers

Details of the drugs and markers used are listed in Table 1, including doses. Sodium valproate (>98%) was administered in one of four doses: 10, 30, 100 or 200 mg/kg body weight. This covered the range of doses from below to above those used clinically in monotherapy or combined with lamotrigine (T O’Brien and F Vajda, personal communication). Sodium valproate was dissolved in sterile isotonic (0.9%) sodium chloride solution and ^3^H-valproate (for amount see Table 1) was added prior to injection.

Lamotrigine was administered in one of three doses: 6, 20 and 40 mg/kg body weight This covered the range of doses from below to above those used clinically in monotherapy or combined with valproate (T O’Brien, N Jones and F Vajda, personal communication). Lamotrigine was dissolved in 100% ethyl alcohol and ^3^H-lamotrigine was added prior to injection ( Table 1). Four preparations were used for combination therapy experiments. These were intended to achieve blood concentrations in the rat equivalent to the range used in combination therapies clinically in humans (T O’Brien, N Jones and F Vajda, personal communication). For all combination experiments, two injections were used. The first injection always contained 30 mg/kg valproate dissolved in sterile saline. The second injection was one of either 2 mg/kg, 6 mg/kg, 12 mg/kg or 20 mg/kg of lamotrigine dissolved in 100% ethanol. Depending on the entry of which was to be estimated, either ^3^H-valproate was added to the first injection, or ^3^H-lamotrigine was added to the second injection.

The drugs were administered intraperitoneally ( *i.p.*). ^3^H-dextran was administered intravenously for estimation of residual vascular space as described below.

### Chronic experiments with valproate

For chronic experiments, female rats were fed for two weeks prior to mating on a feed premixed with 20 g/kg valproate (Specialty Feeds, Sigma Aldrich). This feed has been shown to achieve consistent blood concentrations of ~220 μmol/L (approaching human clinical range of 300–600 μmol/L) of valproate across a sustained period ( Jazayeri *et al*., 2020). For successfully time-mated females, feeding continued up until the point of fetus or postnatal (P4) sample collection. At termination of the experiment drug entry was estimated as for acute experiments, *i.e*. animals then received an acute injection of 100 mg/kg valproate with ^3^H-valproate, the preparation of which is described below.

### Anaesthesia

All time-mated pregnant females at E19, as well as postnatal animals used for residual vascular space estimations received an *i.p.* injection of 25% *w*/ *v* urethane (1.0 mL/100 g body weight). Postnatal animals in valproate, lamotrigine and combination treatment groups were anaesthetised using inhaled isoflurane. The reason for the difference in anaesthesia was the duration of the experiment and/or route of sample collection, see below. Deep anaesthesia was ensured by unresponsiveness to the pinching of the toe and tail before surgery commenced.

E19: Following deep anaesthesia, pregnant females at E19 were placed on a 39°C heating mat in a supine position. An endotracheal catheter was inserted to provide a clear airway. The femoral artery was cannulated to allow time-matched maternal blood samples to be taken at the time of each individual pup sampling ( Koehn *et al*., 2019). Blood volume loss was approximately replaced with 0.9% sterile sodium chloride solution. Samples were collected from the first fetus 30 min following *i.p.* drug injection, continuing up to 2.5 hours post-injection, at approximately 10 min intervals. Time-matched blood samples were used to estimate placental transfer at the time of each fetal collection, according to Equation 3. Previous experience has shown that fetuses can be maintained in reasonable physiological condition for about 3-4h following induction of anaesthesia.

P4 and adult animals: at both ages the animals underwent similar procedures. 30 min following the *i.p.* injection of drug, the animals were anaesthetised using inhaled isoflurane and the diaphragm was cut to terminate the experiment. Blood was drained from the right ventricle of the heart using a heparinised glass micropipette attached to PVC tubing for P4 animals or a heparinised syringe for adult animals.

### Tissue collection

Blood, CSF, cortex, brainstem, and thymus samples were collected from fetuses, pups and adults. As ABC transporters are a key mechanism of limiting drug entry into the brain and the main such transporter appears to be P-glycoprotein, the thymus was included for comparison, as it is known to have limited expression of this efflux transporter ( Valente *et al*., 2008, and see Discussion). At E19, at the end of each period of exposure to drug the experiment was terminated by exsanguination of the fetus by sampling blood from the right ventricle using heparinised glass micropipettes attached to PVC tubing ( Habgood *et al*., 1993). Maternal blood samples were taken *via* the arterial cannula with a final sample taken from the right ventricle of the heart with a syringe. In all animals, CSF was taken from the cisterna magna, with careful suction through glass micropipettes attached to PVC tubing. CSF was briefly centrifuged and checked under a microscope for blood contamination after collection. As little as 0.2% of blood contamination can be detected in 15–20 μL ( Habgood *et al*., 1992). The left and right cortices of the brain were taken following careful exposure of the lateral ventricles, to avoid contamination from the choroid plexus and CSF, followed by the brainstem. For comparison with postnatal animals, additional experiments were conducted following direct *i.p.* injection to the fetus (sample collected 30 min later) as described for postnatal animals.

### Sample preparation

Blood samples were centrifuged at 5000 rpm for 5 mins to separate the plasma. Measured volumes (not exceeding 20 µL) of plasma, CSF, and injectate were transferred into scintillation tubes. 5 mL of scintillation fluid (Emulsifier-safe, PerkinElmer) was then added. Tissue samples were weighed and up to 50 mg were dissolved in 500 µL of Soluene 350 (PerkinElmer) overnight in a scintillation tube. Two drops of acetic acid were added to each tube to neutralise the alkaline Soluene 350, followed by 5 mL of scintillation fluid (Emulsifier Safe, PerkinElmer). Samples were placed on a liquid scintillation counter (Tri-Carb 4910 TR, PerkinElmer) for 5 mins per sample to count radioactivity disintegrations per minute (DPM). Blank samples taken from rats with no radioactivity were also counted with each run to establish background levels. These were always subtracted from the plasma, CSF or tissue sample counts.

### Extraction and liquid chromatography coupled to mass spectrometry (LC–MS) analyses of valproate

Valproic acid and D6-valproic acid (Novachem, Aus) were dissolved in water at 5 µg/µL as stock. All stock solutions were stored at -20 ℃. The internal standard mixture containing 12 or 120 ng/µL D6-valproic acid in methanol was prepared from diluting stock solutions using methanol immediately before use. 10 µL of internal standard mixture and 80 µL methanol were added to 10 µL plasma, CSF or brain homogenate. Approximately 20 mg of brain tissue were minced and homogenised in a volume of deionized water (in µL) equal to twice the weight of the tissue using a glass homogeniser; 10 µL of the homogenate was used for each sample. After vortexing for 30 seconds, the sample was centrifuged at 14,000× *g* for 10 min and the top 50 µL was transferred to glass vial for liquid chromatography coupled to mass spectrometry (LC–MS) analysis using a Vanquish ultrahigh performance liquid chromatography (UHPLC) linked to an Orbitrap Fusion Lumos mass spectrometer (Thermo Fisher Scientific, San Jose, CA, USA) operated at positive ion mode. Solvent A was 10 mM ammonium formate with 0.1% formic acid in water and solvent B was acetonitrile. 25 µL of each sample was injected into an RRHD Eclipse Plus C18 column (2.1×1000 mm, 1.8 µm; Agilent Technologies, Santa Clara, CA, USA) at 50°C at a flow rate of 350 μL/min for 1 min using 5% solvent B. During separation, the percentage of solvent B was increased from 5% to 40% in 4 min. Subsequently, the percentage of solvent B was increased to 80% in 0.5 min and then maintained at 99% for 2 min. Finally, the percentage of solvent B was decreased to 5% in 0.1 min and maintained for 2.4 min.

All MS experiments were performed using a heated electrospray ionization (HESI) source. The spray voltage was 3.0 kV in negative ionisation mode. The flow rates of sheath, auxiliary and sweep gases were 20 and 6 and 1 arbitrary unit(s), respectively. The ion transfer tube and vaporizer temperatures were maintained at 350°C and 400°C, respectively, and the S-Lens RF level was set at 50%. The full-scan MS-spectra were acquired in the Orbitrap at a mass resolving power of 120,000 (at m/z 200) across an m/z range of 100–1000 using quadrupole isolation, auto-gain control (AGC) target of 4E5, auto maximum injection time at both polarities. Targeted higher-energy collisional dissociation (HCD)-tandem mass spectrometry (MS/MS) scan of valproic acid at m/z 143.1072 and D6-valproic at m/z 149.1394 acid were performed with normalized collision energy (NCE) at 0%, isolation width of 4 Da, Orbitrap resolution at 15,000 (at m/z 200), maximum injection time of 22 milliseconds and AGC target of 2.5E5. Ion chromatogram peak area of ions in MS/MS scans at m/z 143.1072 from valproic acid and 149.1394 from D6-valproic acid at 6.6 min were extracted using Skyline 20.2 (RRID:SCR_014080) to calculate the concentration of drug in each sample. The linear response range of valproic acid in different sample types tested for the LC–MS/MS analysis is provided in the extended data ( Toll *et al.*, 2021). 

### Data calculations and statistical analysis

All radioactivity counts were normalised to weight/volume and expressed as DPM/mg or μL of sample. The residual vascular space for each tissue sample was calculated ( Equation 1). These values were then used to correct tissue counts with results expressed as tissue/ or fluid/plasma ratios % ( Equation 2). Fetal and maternal blood samples at E19 were used to obtain placental transfer ( Equation 3).

Equation 1


$$Residual\;vascular\;space\;=\;\frac{Tissue\;DPM/\mu L}{Plasma\;DPM/\mu L}\;\times \;100\%$$

Equation 2


$$CSF\;or\;tissue\;entry\;=\;\frac{CSF\;or\;tissue\;DPM/\mu L}{Plasma\;DPM/\mu L}\;\times \;100\%-RVS\%$$

Equation 3


$$Placental\;transfer\;=\;\frac{Fetal\;plasma\;DPM/\mu L}{Maternal\;plasma\;DPM/\mu L}\;\times \;100\%$$

Data from all experiments are expressed as mean±standard deviation (SD). Statistical differences across time-course experiments, dose experiments with greater than two doses, combination experiments with greater than two doses and age comparisons were obtained using a one-way ANOVA with Tukey’s multiple comparisons test. Differences between dose experiments with two treatments, combination experiments with two treatments, and comparisons between acute and chronic groups were obtained using an unpaired two-tailed t-test. All tests were completed using Prism (GraphPad Software Inc; RRID:SCR_002798) with p<0.05 accepted as statistically significant. All statistical analyses can be performed using JASP (RRID:SCR_015823), an open-source alternative.

## Results

Most results are expressed as tissue or CSF to plasma concentration ratios (equations 1–3 above). This is an established way to represent the entry (permeability) of markers that cross cellular interfaces as this takes into account variability in marker concentrations in plasma due to variations in *in vivo* experimentation ( Davson & Segal, 1996)

### Estimation of residual vascular space

**Figure 1.**
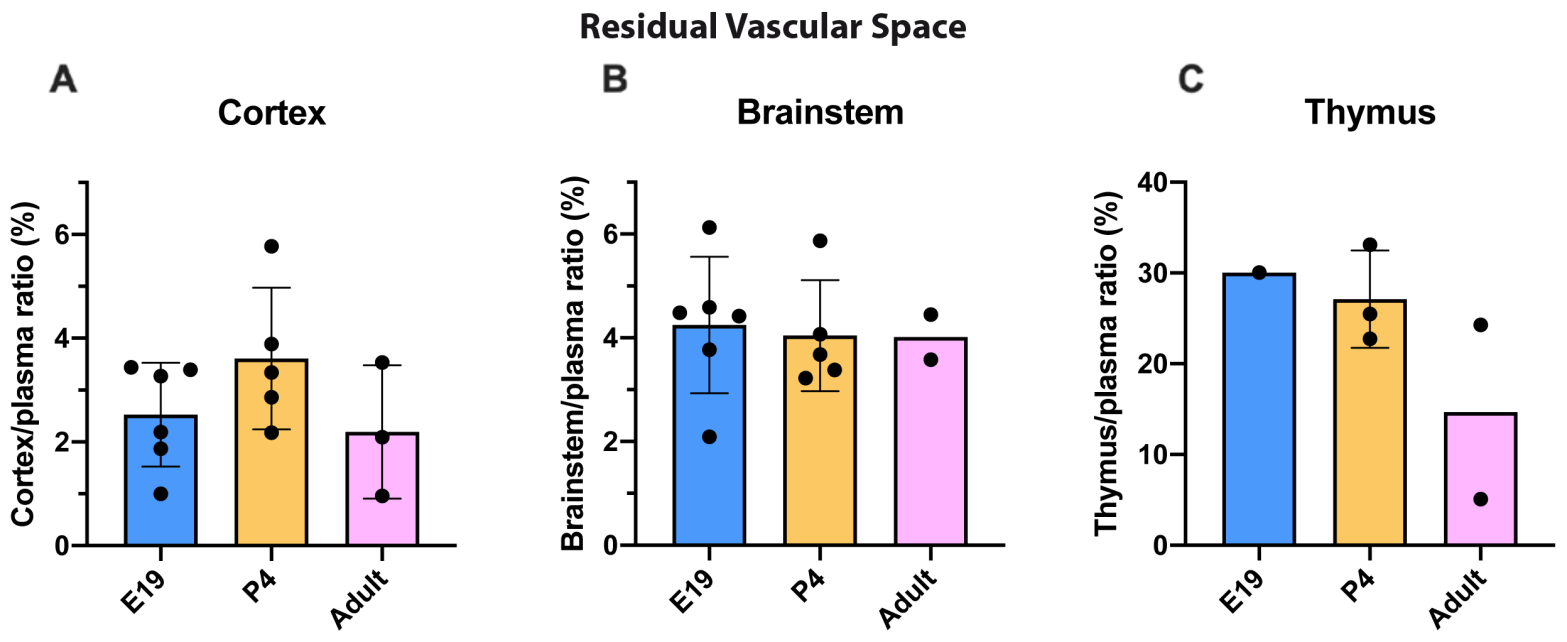
Estimation of residual vascular space. **A**. Cortex/plasma, **B**. Brainstem/plasma and **C**. Thymus/plasma concentration ratios (%) of ^3^H-dextran in rats at E19, P4 and in non-pregnant female adults, collected 5 minutes after a single intravenous injection of radioactive tracer ( ^3^H-dextran). Each point represents the result from a single animal. Mean±standard deviation (SD). E19; n=6, except for thymus where a single sample was obtained by pooling (1 litter), P4; n=3–5, adult; n=2–3. Note different scale in **C**.

Residual vascular space in the brain was estimated using 70 kDa dextran radiolabelled with ^3^H which was injected *i.v.* and left to circulate for only 5 min. The concentration ratio of the dextran in the brain tissue compared with plasma was used as an indicator of the residual blood space, as such a large molecule is not expected to leave the circulation in 5 min only ( Habgood *et al*., 1993). The ratio of ^3^H-dextran into cortex and brainstem compared with plasma, illustrated in Figure 1A and B, showed no significant difference at any of the ages studied and was around 2–4%. In contrast, thymus residual vascular space appeared to be greater at younger ages (E19 and P4) than in the adults but samples were too limited in numbers (due to the necessity of pooling) for this to be tested formally ( Figure 1C).

All results for brain and thymus drug permeability studies described subsequently have been corrected for residual vascular space. CSF was checked for blood contamination as described in the Methods. Four contaminated samples were discarded.

### Time-dependent entry of valproate and lamotrigine in P4 rats

**Figure 2.**
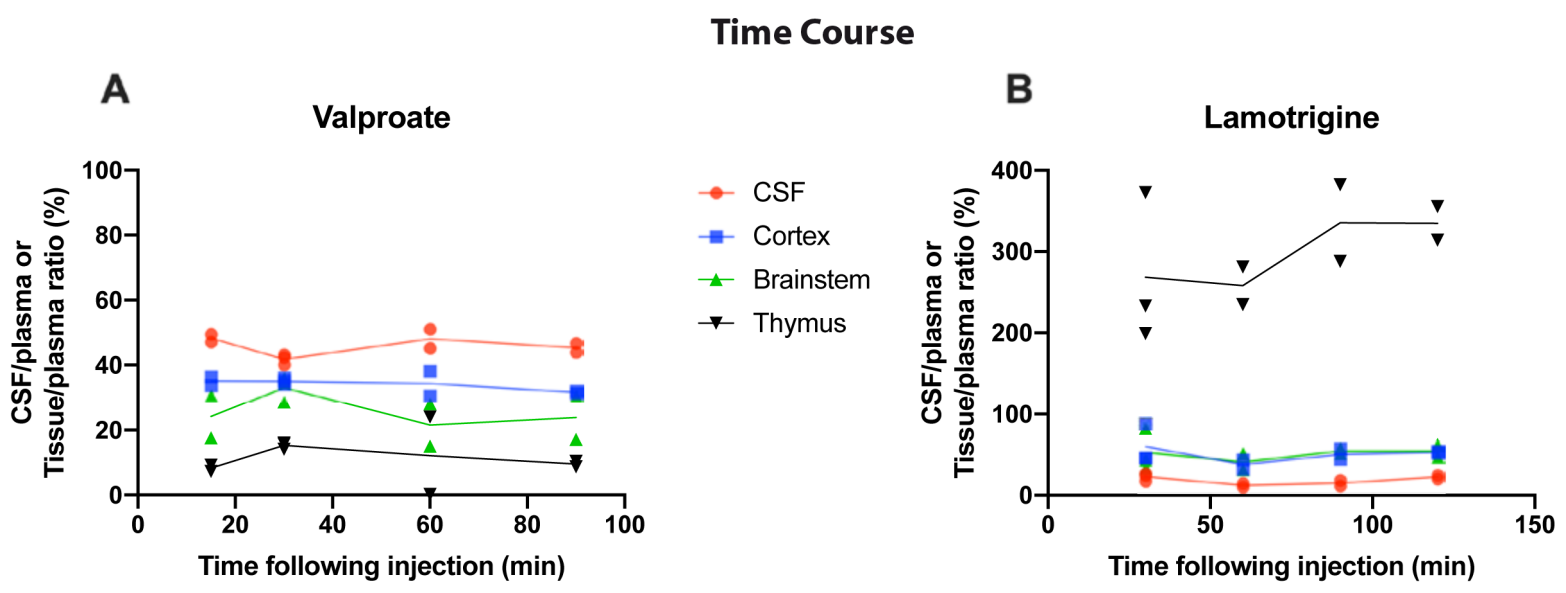
Time-course of valproate and lamotrigine CSF and tissue entry in P4 rats. CSF/plasma, Cortex/plasma, Brainstem/plasma, and Thymus/plasma concentration ratios (%) of **A**. Valproate (single intraperitoneal injection of 100 mg/kg valproate with radioactive tracer ^3^H-valproate) and **B**. Lamotrigine (single intraperitoneal injection of 20 mg/kg of lamotrigine with ^3^H-lamotrigine). Samples were collected between 15 and 120 min after injection. Each point represents the result from a single animal.

To establish if entry of valproate and lamotrigine into the brain and CSF changed depending on the duration of exposure to the drug, time course experiments were conducted at P4. Results are shown in Figure 2. Following a single *i.p.* injection, the entry of ^3^H-valproate into the CSF, cortex, brainstem, and thymus of P4 rats was similar between 15 min and 90 min, as was also the case for entry of ^3^H-lamotrigine between 30 min and 120 min ( Figure 2); therefore, all subsequent acute experiments were performed 30 min following *i.p.* injection, as in previous similar experiments ( Koehn *et al*., 2019).

### Dose-dependent entry of valproate and lamotrigine

**Figure 3.**
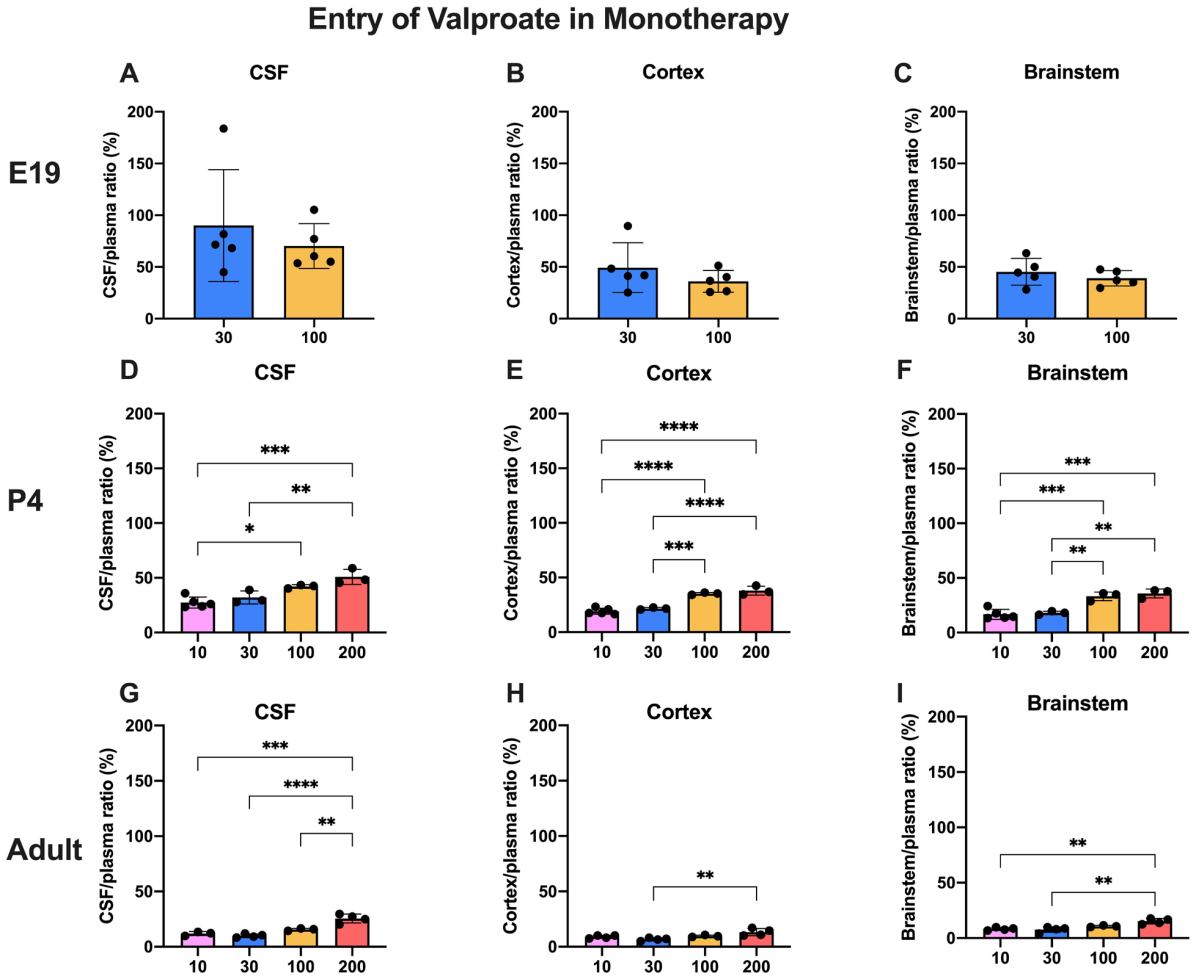
Dose response entry of valproate into CSF and brain. CSF/plasma, Cortex/plasma and Brainstem/plasma concentration ratios (%) in E19 ( **A**– **C**), P4 ( **D**– **F**) and non-pregnant female adult ( **G**– **I**) rats, collected 30 minutes after a single intraperitoneal injection of valproate with radioactive tracer ( ^3^H-valproate). Doses in mg/kg indicated on the x-axis. Each point represents the result from a single animal. Mean±SD; n=3-5. *p<0.05, **p<0.01, ***p<0.001, ****p<0.0001. Note that some error bars are too small to be clearly visible.

The doses of valproate and lamotrigine and the ages at which they were studied are shown in Table 1. The entry of ^3^H-valproate into the CSF, cortex and brainstem following *i.p.* injection into the fetuses, postnatal animals or adults are shown in Figure 3

Transfer into the brain and CSF at E19 was not significantly different between the two doses tested (30 mg/kg and 100 mg/kg). This indicates that drug concentration in this range had little influence on the permeability of valproate across the blood–brain and CSF–brain barriers at E19. Actual values for CSF/plasma and tissue/plasma ratios are shown in extended data Table 3A.

At P4, the entry of 30 and 100 mg/kg valproate was appreciably less than at E19 and this was even more the case in the adult ( Figure 3). However, there was no difference in entry of these two doses at P4 or in adults. At the higher doses (100 and 200 mg/kg) the CSF and brain/plasma ratios were significantly higher than for the lower doses (actual values shown in the extended data Table 3A), indicating a dose-dependent relationship of valproate entry in postnatal pups and in adults. In the adult the entry of the highest dose (200 mg/kg) was nevertheless still substantially below that of the lower doses at E19 ( Figure 3).

**Figure 4.**
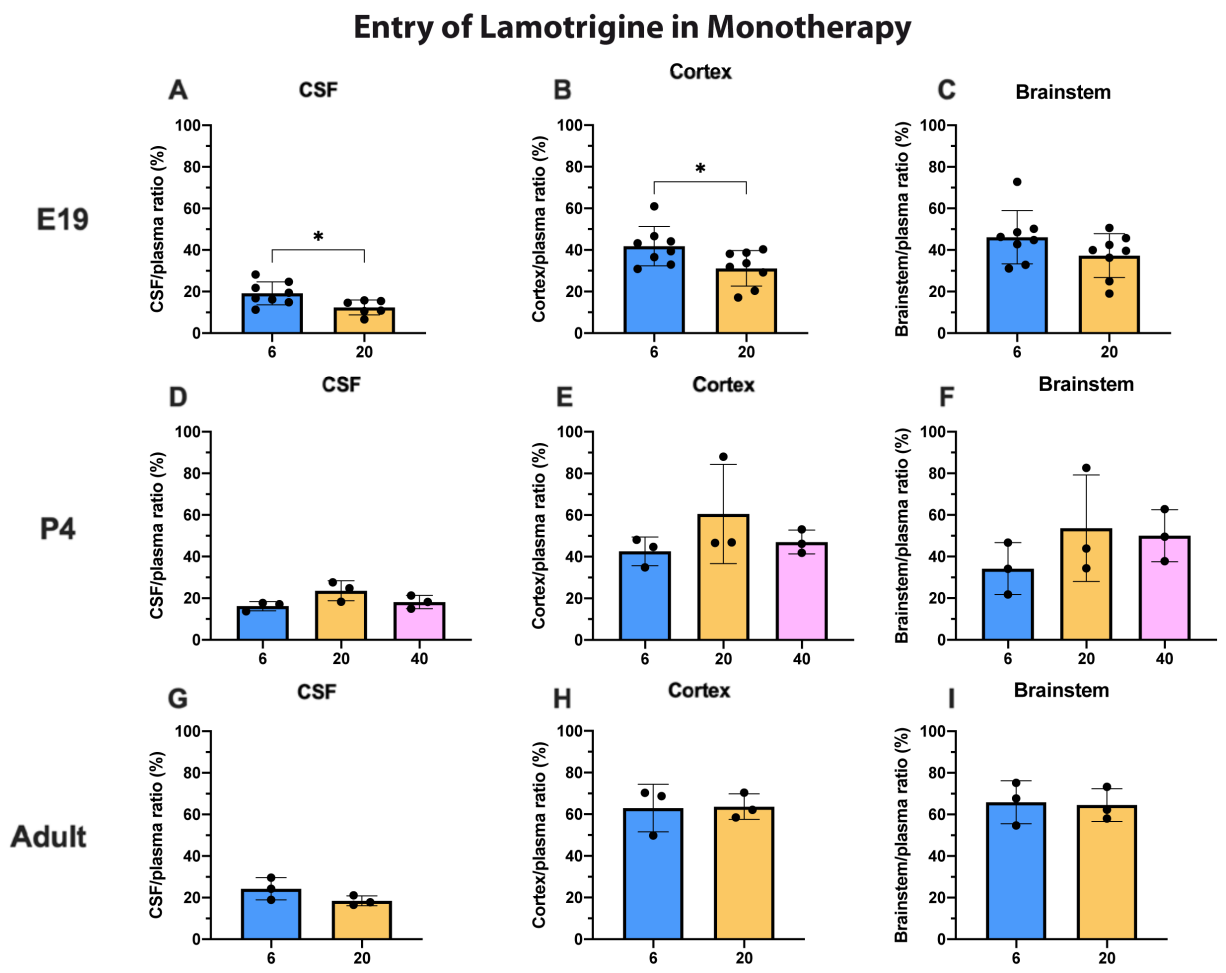
Dose response entry of lamotrigine into CSF and brain. CSF/plasma, Cortex/plasma and Brainstem/plasma ratios (%) in E19 ( **A**– **C**), P4 ( **D**– **F**) and non-pregnant female adult ( **G**– **I**) rats, collected 30 minutes after a single intraperitoneal injection of lamotrigine with radioactive tracer ( ^3^H-lamotrigine). Doses in mg/kg indicated on the x-axis. Each point represents the result from a single animal. Mean±SD; n=3–12. *p<0.05. Note that some error bars are too small to be clearly visible.

^3^H-lamotrigine entry into the CSF, cortex and brainstem at all three ages is illustrated in ( Figure 4). The only significant differences were for CSF and cortex values at E19 when the entry following injection of 20 mg/kg was marginally less (p<0.05). In general, permeability across the blood–brain and blood–CSF barriers did not appear to be dose-dependent over the concentration range studied at P4 and in adults and only marginally so at E19. Actual values for CSF/plasma and tissue/plasma ratios are in the extended data Table 4A.

### Age-dependent entry of valproate and lamotrigine

**Figure 5.**
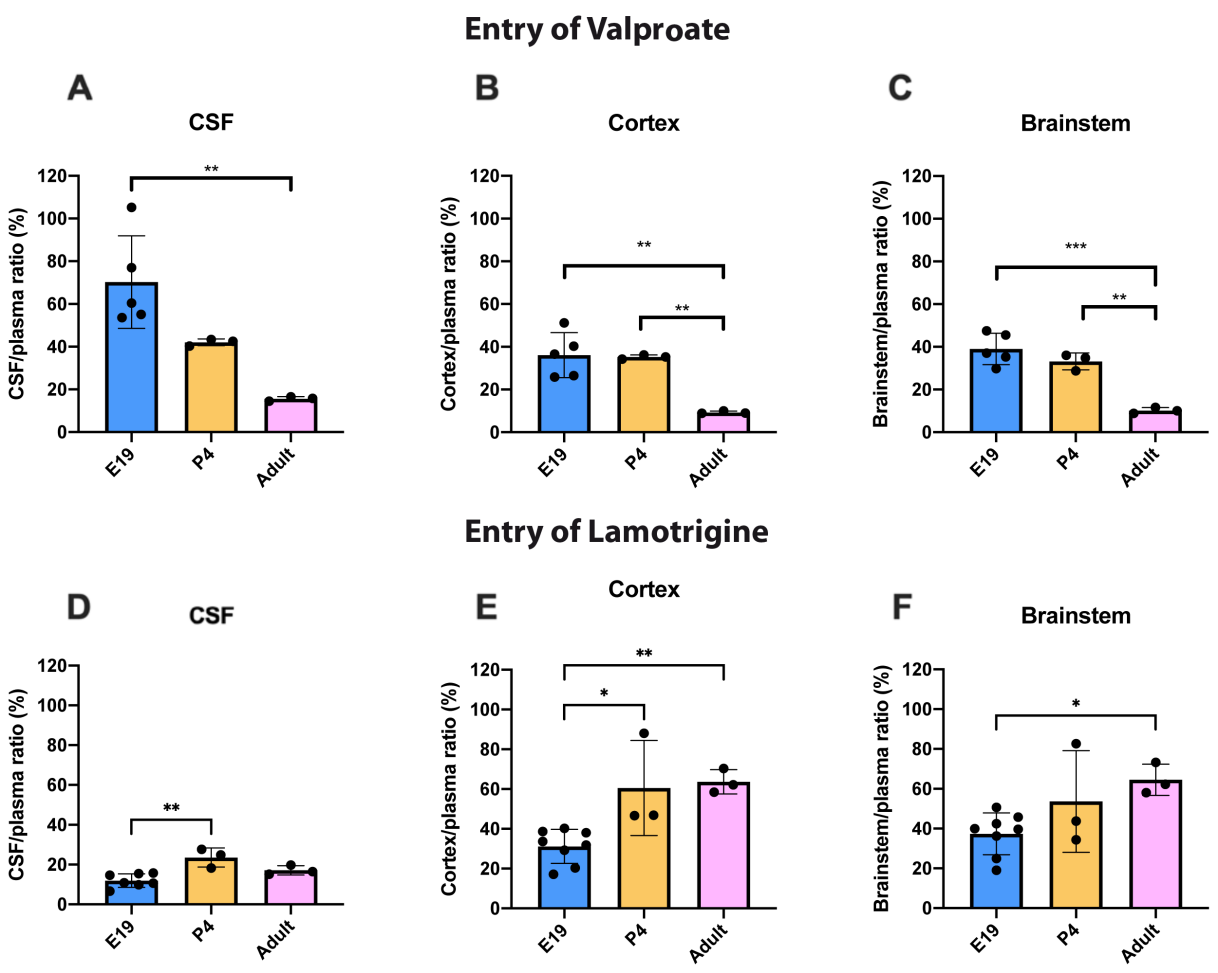
Age comparison of drug entry into CSF and brain. CSF/plasma, Cortex/plasma and Brainstem/plasma concentration ratios (%) of valproate ( **A**– **C**) or lamotrigine ( **D**– **F**) at E19, P4 and in non-pregnant female adult rats collected 30 min after a single intraperitoneal injection of 100 mg/kg valproate or 20 mg/kg lamotrigine respectively. Each point represents the result from a single animal. Mean±SD. n=3–8. *p<0.05, ** p<0.01. Note that some error bars are too small to be clearly visible.

To illustrate the age dependence of drug entry into CSF and brain data from Figure 3 and Figure 4 have been replotted in Figure 5 for 100 mg/kg valproate and 20 mg/kg lamotrigine. For ^3^H-valproate, there was little difference between E19 and P4, but the adult values were substantially and significantly lower (p<0.01–0.001). For 20 mg/kg lamotrigine entry into CSF was significantly greater at P4 compared with E19 (p<0.01). There were also increases in brain entry between E19 and adults (p<0.05–0.01). Values of CSF/plasma and tissue/plasma are in the extended data Table 5A.

### Entry of valproate and lamotrigine in combination therapy

**Figure 6.**
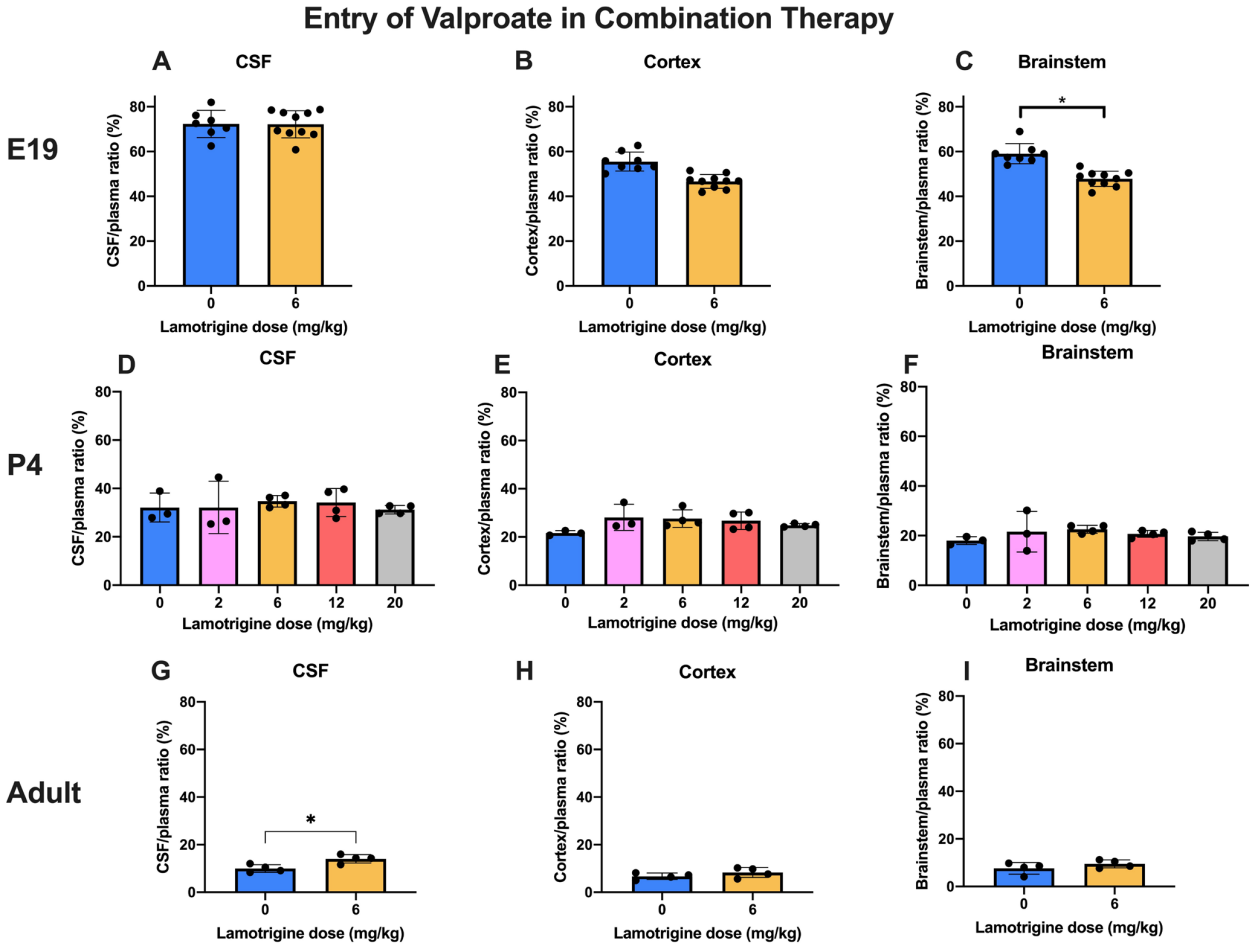
Valproate entry into CSF and brain in combination therapy. All animals received 30 mg/kg of valproate with radioactive tracer ( ^3^H-valproate) as a monotherapy, or in combination with different doses of lamotrigine. CSF/plasma, Cortex/plasma and Brainstem/plasma concentration ratios (%) in E19 ( **A**– **C**), P4 ( **D**– **F**) and non-pregnant female adult ( **G**– **I**) rats of valproate, collected 30 min after intraperitoneal injections. Each point represents the result from a single animal. Mean±SD. n=3–10. *p<0.05. Note that some error bars are too small to be clearly visible.

***Valproate.*** In these experiments, entry of ^3^H-valproate in the presence of increasing doses of lamotrigine (0–6 mg/kg at E19 and in adults and up to 20 mg/kg at P4) was estimated ( Figure 6). Even over the extended dose range at P4 there were no differences in CSF or brain/plasma ratios. At E19, there was a small but significant (p<0.05) decrease in the brainstem entry with the 6 mg/kg dose. In the adult there was a small but significant (p<0.05) increase in entry into CSF with the 6 mg/kg dose. Values for CSF/plasma and tissue/plasma ratios are in Supplementary Material Table 6A.

**Figure 7.**
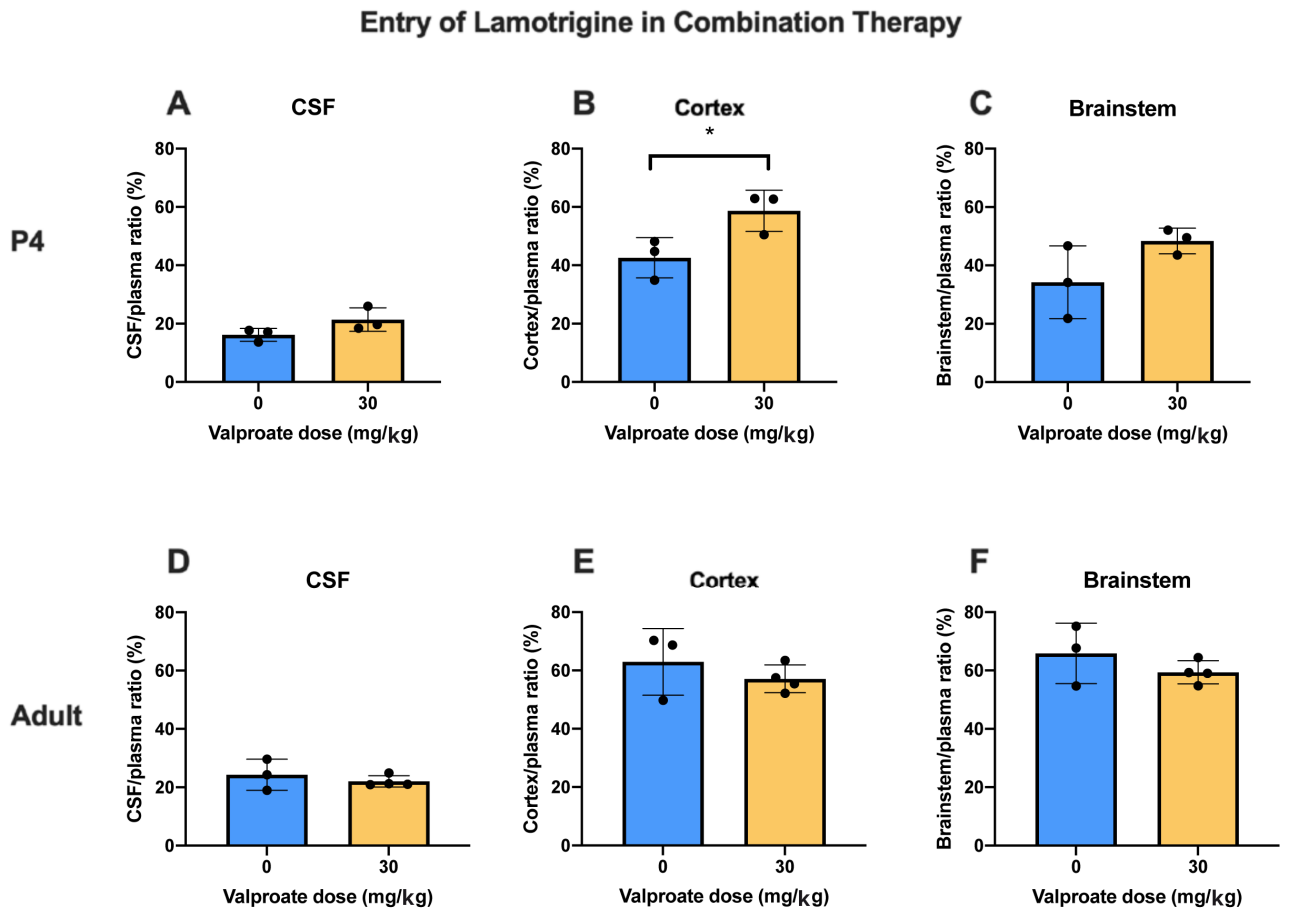
Lamotrigine entry into CSF and brain in combination therapy. All animals received 6 mg/kg of lamotrigine with radioactive tracer ( ^3^H-lamotrigine) as a monotherapy, or in combination with 30 mg/kg of valproate. CSF/plasma, Cortex/plasma and Brainstem/plasma concentration ratios (%) in P4 ( **A**– **C**) and non-pregnant female adult ( **D**– **F**) rats of lamotrigine, collected 30 min after intraperitoneal injections. Each point represents the result from a single animal. Mean±SD. n=3–4. *p<0.05.

***Lamotrigine.*** In the converse experiment P4 and adult animals were injected *i.p*. with 6 mg/kg lamotrigine with ^3^H-lamotrigine, alone or with 30 mg/kg valproate ( Figure 7). The only significant difference (p<0.05) was at P4 in the cortex. Otherwise the addition of valproate to lamotrigine did not appear to influence the entry of lamotrigine. Values for CSF/plasma and tissue/plasma ratios are in the extended data Table 7A.

### Chronic treatment with valproate

Animals assigned to the chronic group were treated with a feed containing 20 g/kg valproate (see Methods and Jazayeri *et al*., 2020) for at least 3 weeks for non-pregnant adults, and at least 2 weeks prior to mating followed by a further 19 days for E19 experiments and 25–26 days for P4 experiments. They were compared with animals fed chronically with food not containing valproate. Thus E19 pups received the drug exclusively *via* placental transfer and following birth P4 animals continued to receive valproate *via* breastmilk. All chronically fed animals and controls were then treated with the same protocol as the acutely treated animals (see Methods) that received an injection of 100 mg/kg valproate containing a radioactive ^3^H-valproate 30 min prior to sample collection.

**Figure 8.**
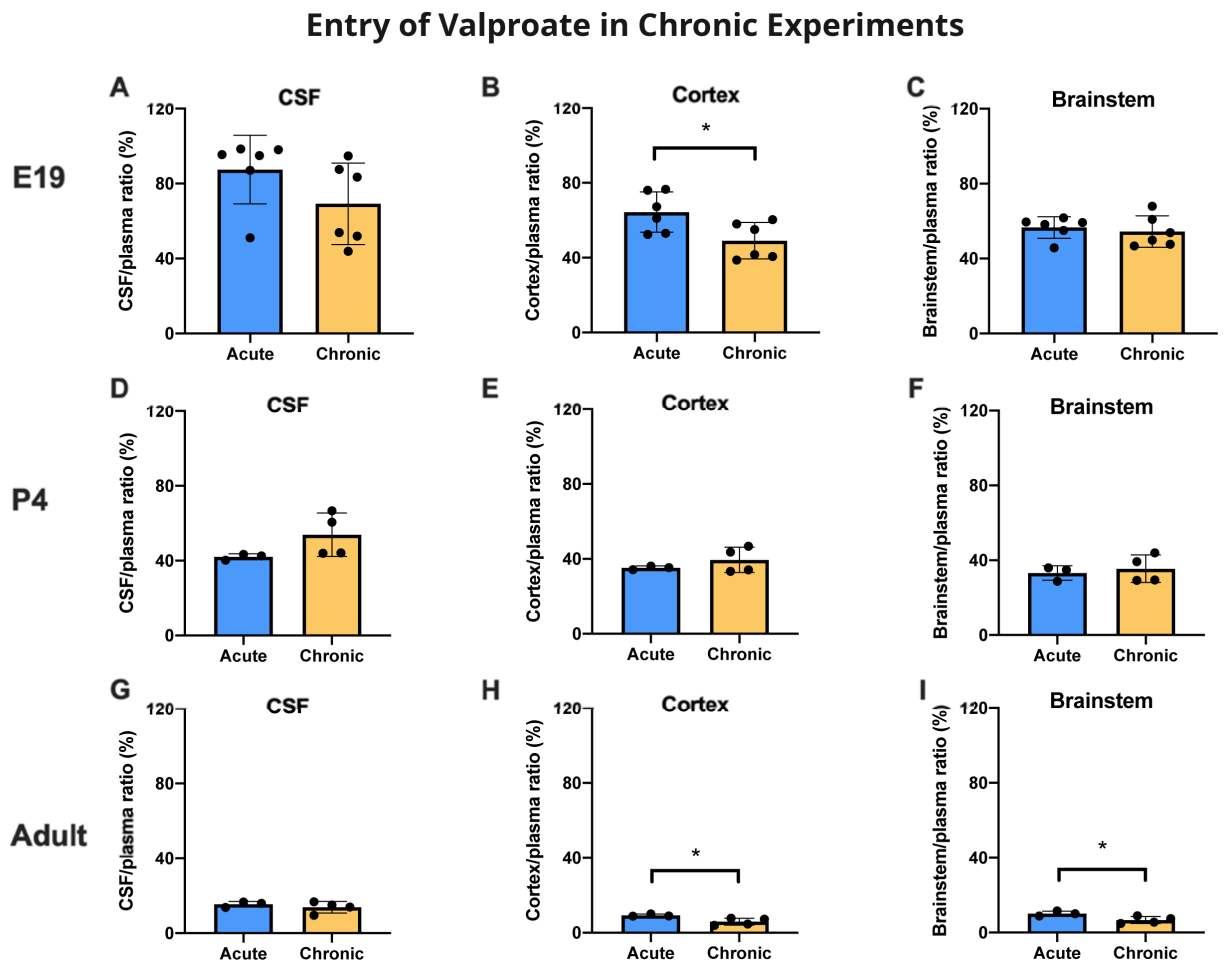
CSF and brain entry of valproate with or without chronic drug feeding. CSF/plasma, Cortex/plasma and Brainstem/plasma concentration ratios (%) in E19 ( **A**– **C**), P4 ( **D**– **F**) and non-pregnant female adult ( **G**– **I**) rats of valproate, collected 30 min after intraperitoneal injections of 100 mg/kg valproate with radioactive tracer ( ^3^H-valproate). Dams of the chronic group were treated with a special feed containing valproate for at least two weeks prior to mating whereas dams of the acute group received a control feed. Non-pregnant female adult rats in the chronic group were treated with a special feed containing valproate for at least three weeks prior to experimentation. Each point represents the result from a single animal. Mean±SD. n=3–6. *p<0.05. Note that some error bars are too small to be clearly visible.

Entry of ^3^H-valproate into the cortex of E19 fetuses following chronic feeding of the mother was significantly lower (p<0.05) than in the fetuses of the acutely treated mother ( Figure 8). At this age, there were no significant differences for CSF or brainstem for acutely and chronically treated animals ( Figure 8). At P4, there were no significant differences for CSF or brain. In the adult there were small but statistically significant (p<0.05) differences for both brain regions in which ratios were slightly lower in the chronically fed animals. In adult animals the entry of valproate in those fed chronically with chow containing valproate or in those on drug free chow was substantially below that in the younger animals. Values for CSF/plasma and tissue/plasma ratios are in the extended data Table 8A.

### Placental transfer

**Figure 9.**
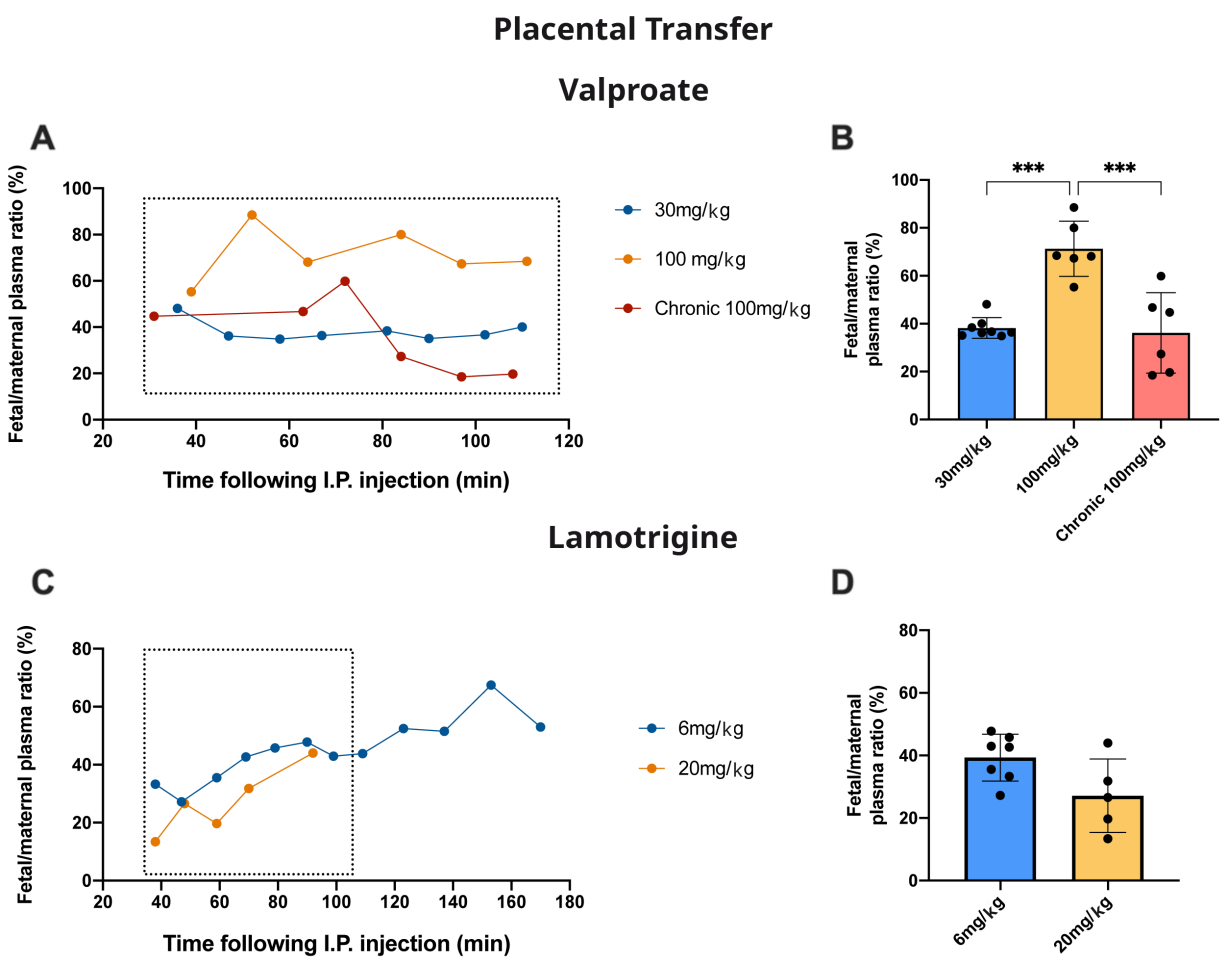
Placental drug transfer. **A**. Time-course of fetal plasma/maternal plasma concentration ratios (%) of valproate collected 30–110 min after an intraperitoneal injection to the mother of valproate with radioactive tracer ( ^3^H-valproate). **B**. Results from **A** are mean±SD. **C**. Time-course of fetal plasma/maternal plasma concentration ratios (%) of lamotrigine collected 40–170 min after an intraperitoneal injection to the mother of lamotrigine with radioactive tracer ( ^3^H-lamotrigine). **D** Results from **C** are mean±SD. For **B** and **D** results were taken up to 110 min (box) to be comparable between experiments. Each point represents the result from a single animal; n=5–12. ***p<0.001.

Measurement of ^3^H-labelled valproate and lamotrigine in maternal and fetal plasma following maternal drug administration allowed an estimate of the level of protection provided by the placenta against drug entry from the maternal circulation into the fetus. At E19, pregnant dams were injected with either valproate or lamotrigine as described for acute experiments. Thirty minutes later samples began to be serially taken from individual fetuses at the times indicated in Figure 9A (valproate) and Figure 9C (lamotrigine) together with time-matched maternal blood samples. In one experiment, the rat was fed with food containing 100 mg/kg valproate from 2 weeks before pairing up to E19 (see Methods). Figures 9A and 9C also show the fetal/maternal ratios for the individual pups. Figures 9B and 9D show the aggregated data up to 110 min. For valproate, in fetuses that received only a single dose of 100 mg/kg valproate, the transfer of the drug expressed as fetal/maternal plasma ratio was significantly higher (p<0.001) than values obtained following either the 30 mg/kg single dose or the chronic 100 mg/kg dose. For lamotrigine there was no significant difference between the two doses administered.

### Entry into thymus

Thymus tissue was collected in this study because it is known to contain little expression of the ABC efflux transporter P-glycoprotein, Abcb1 ( Valente *et al*., 2008) which is the most studied of the efflux mechanisms that exclude or limit the entry of many drugs at brain barriers and for which there is some evidence that lamotrigine may be a substrate ( Zhang *et al*., 2012, see below).

**Figure 10.**
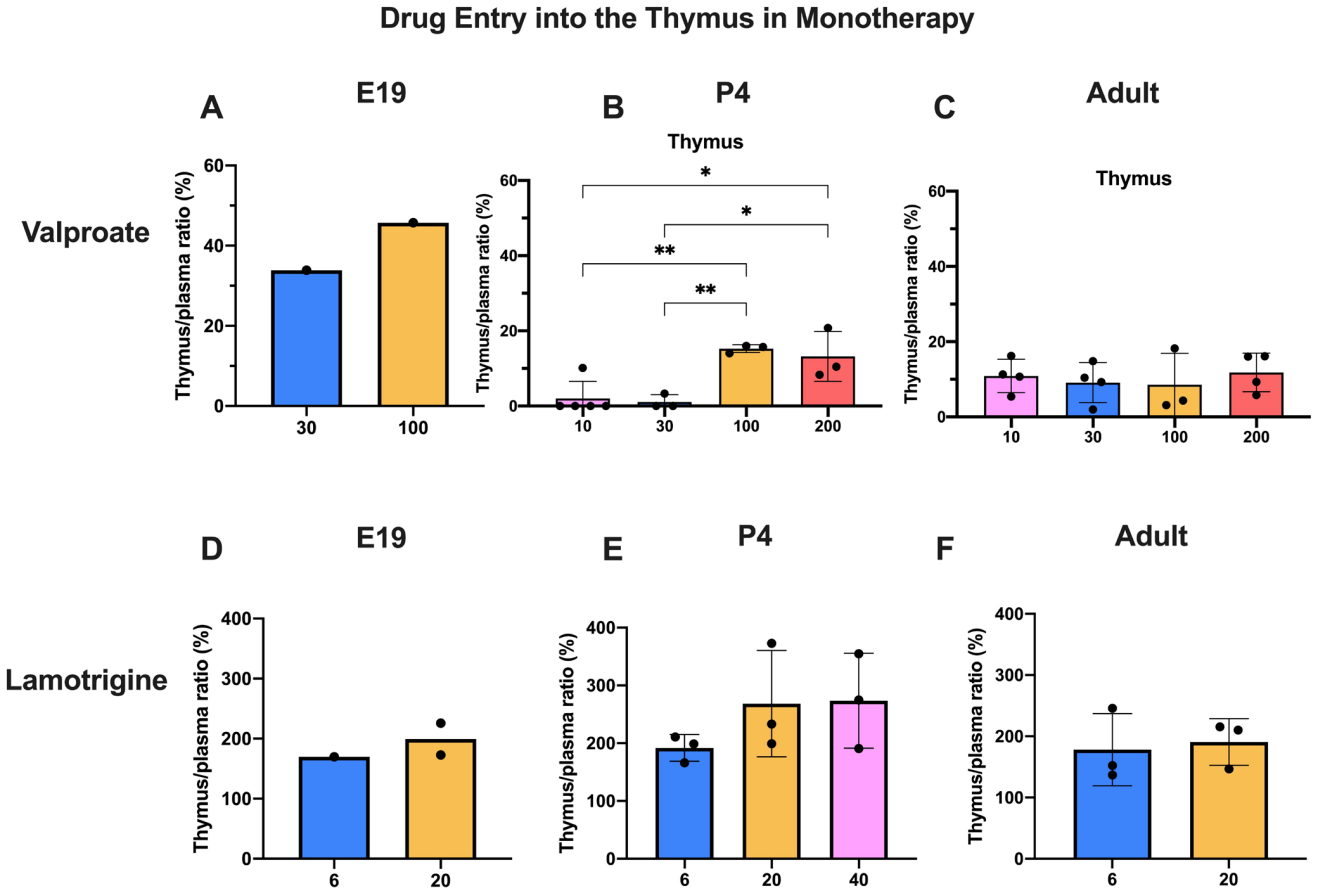
Dose response entry of valproate and lamotrigine into the thymus. Thymus/plasma concentration ratios (%) of valproate ( **A**– **C**) or lamotrigine ( **D**– **F**) at E19, P4 and in non-pregnant female adult rats collected 30 minutes after a single intraperitoneal injection of either drug. Doses in mg/kg indicated on the x-axis. Each point represents the result from a single animal except for E19 thymus where a single sample was obtained by pooling (1 litter). Mean±SD; n=3–4. *p<0.05, **p<0.01. Note that some error bars are too small to be clearly visible.

**Figure 11.**
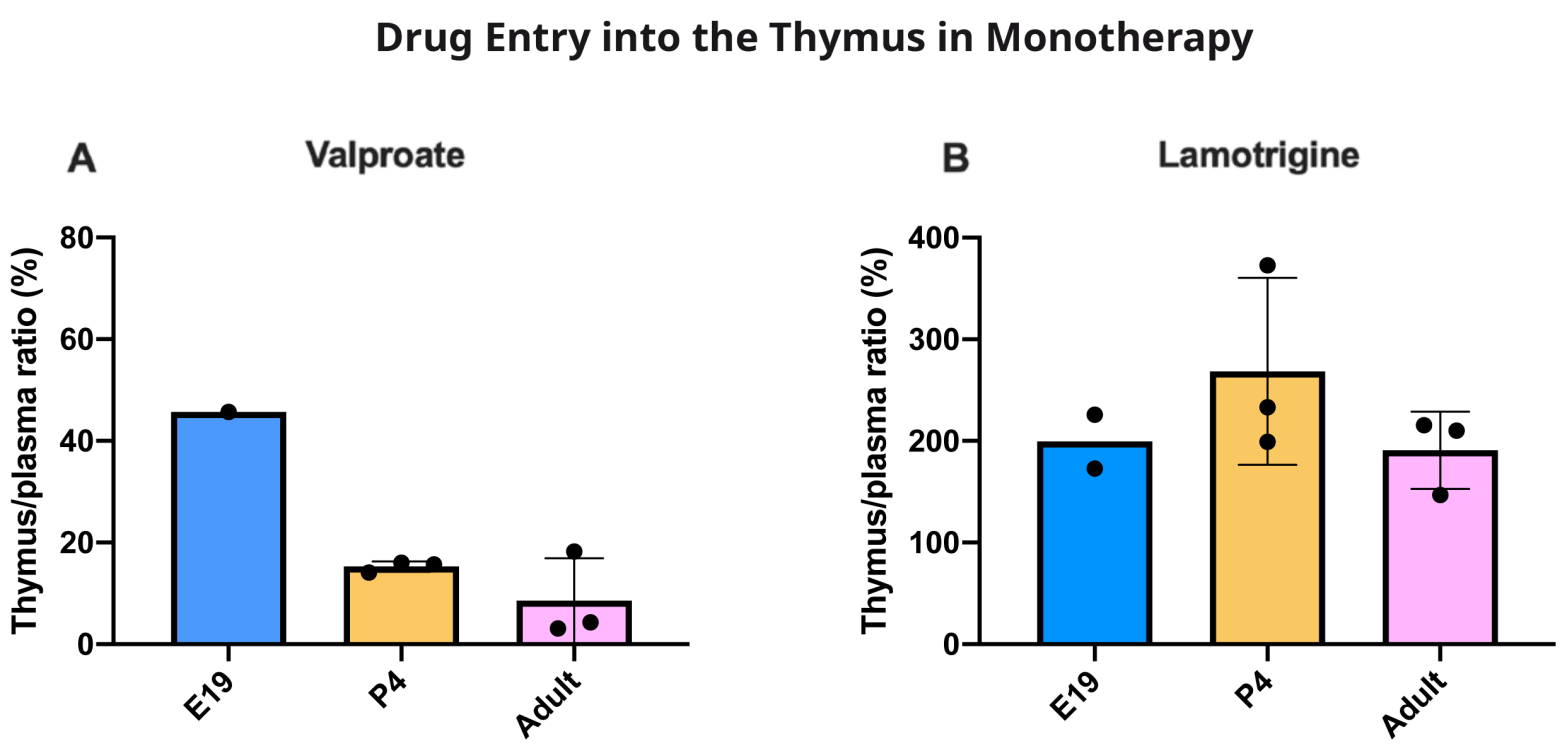
Age comparison of drug entry into the thymus. Thymus/plasma concentration ratios (%) of valproate ( **A**) or lamotrigine ( **B**) at E19, P4 and in non-pregnant female adult rats collected 30 minutes after a single intraperitoneal injection of 100 mg/kg valproate or 20 mg/kg lamotrigine respectively. Each point represents the result from a single animal except for E19 thymus where a single sample was obtained by pooling (1–2 litters). Mean±SD; n=3.

**Figure 12.**
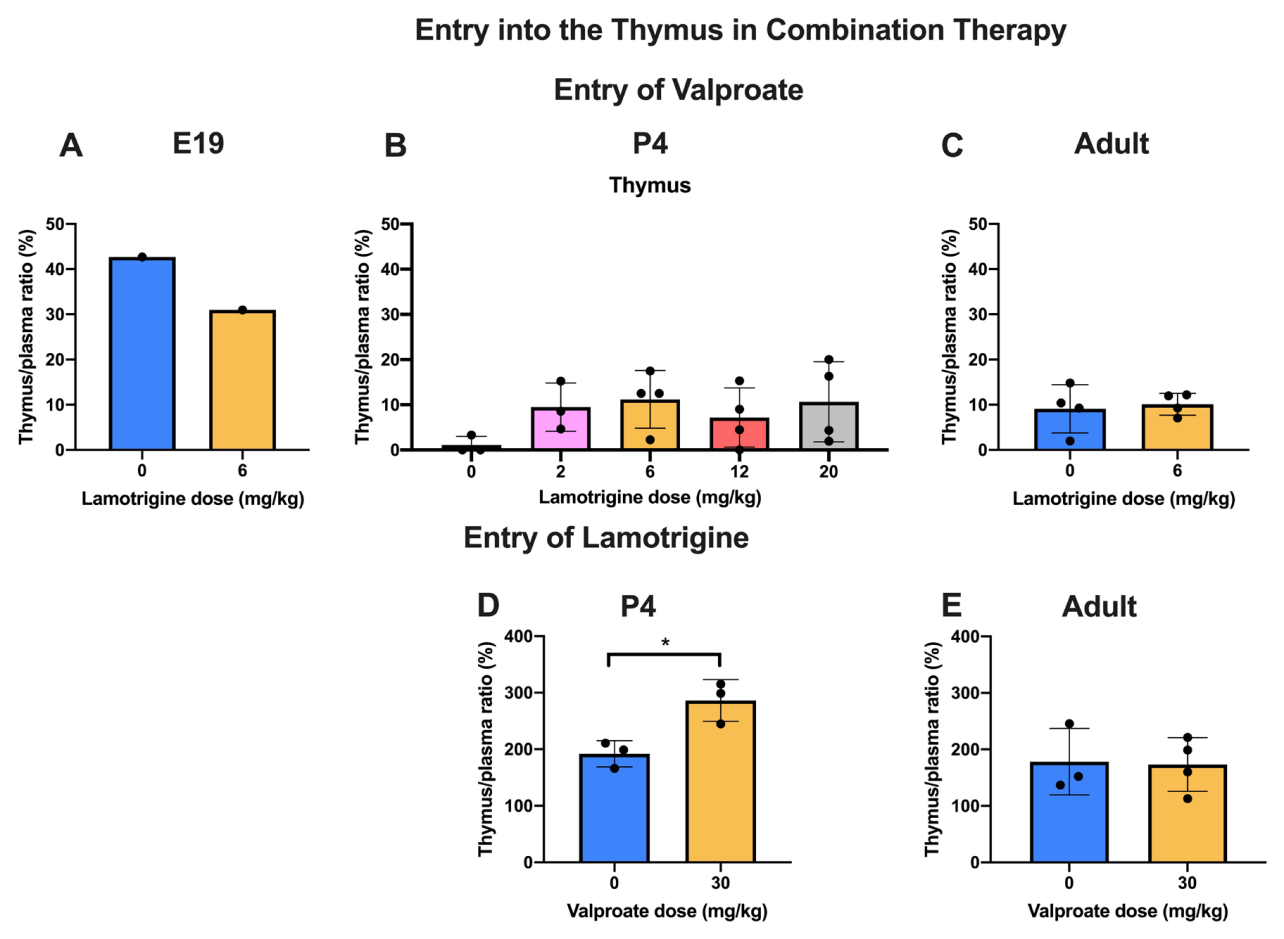
Valproate and lamotrigine entry into the thymus in combination therapy. Thymus/plasma concentration ratios (%) of valproate at E19 ( **A**), P4 ( **B**) or non-pregnant female adult rats ( **C**) collected 30 min after intraperitoneal injections of 30 mg/kg of valproate with radioactive tracer ( ^3^H-valproate) as a monotherapy, or in combination with different doses of lamotrigine. Thymus/plasma concentration ratios (%) of lamotrigine at P4 ( **D**) or non-pregnant female adult rats ( **E**) collected 30 min after intraperitoneal injections of 6 mg/kg of lamotrigine with radioactive tracer ( ^3^H-lamotrigine) as a monotherapy, or in combination with 30 mg/kg of valproate. Each point represents the result from a single animal except for E19 thymus where a single sample was obtained by pooling (1 litter). Mean±SD; n=3–4. *p<0.05.

**Figure 13.**
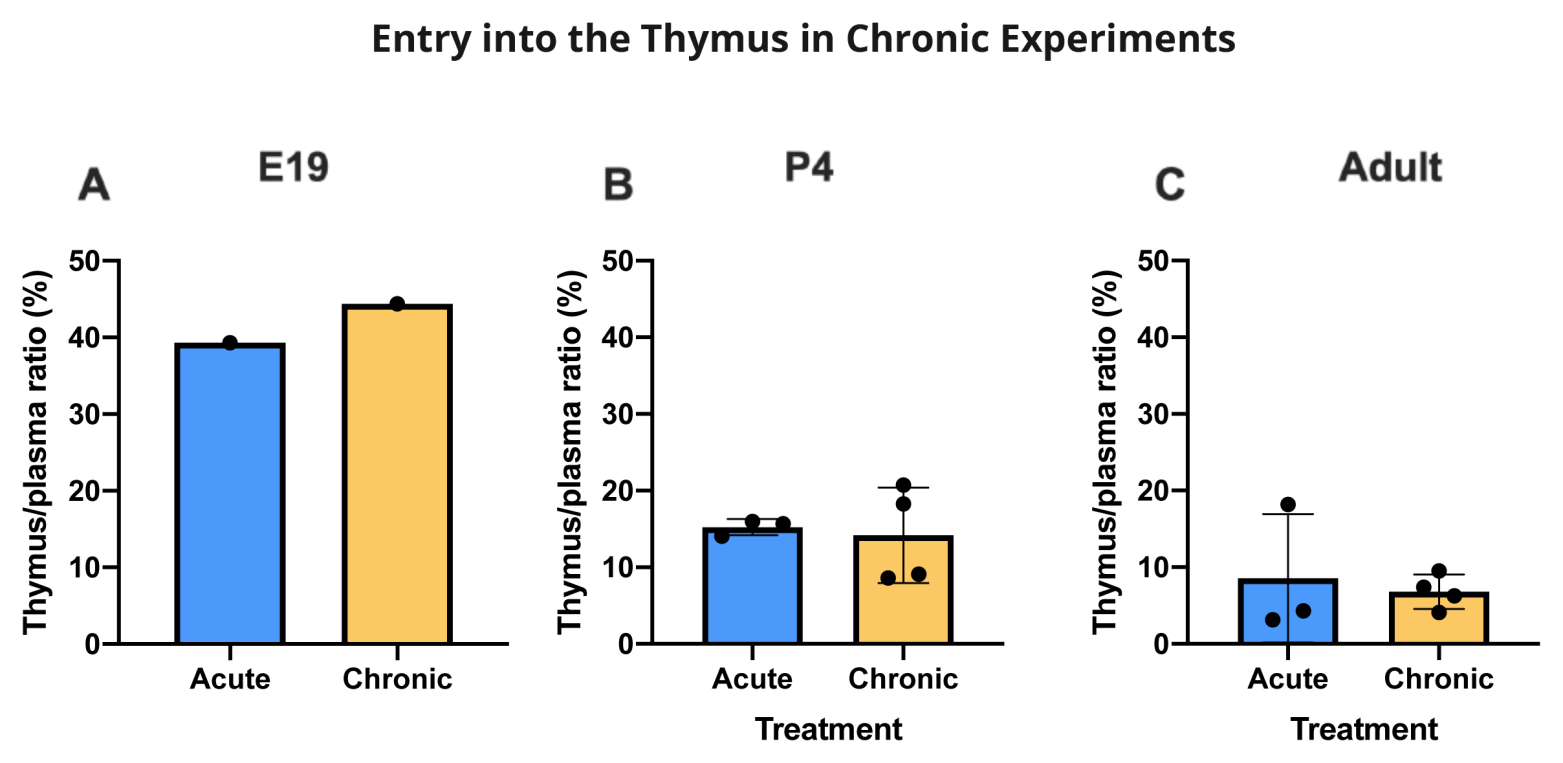
Entry of valproate into the thymus with or without chronic drug feeding. Thymus/plasma concentration ratios (%) of valproate at E19 ( **A**), P4 ( **B**) or non-pregnant female adult rats ( **C**) collected 30 min after intraperitoneal injections of 100 mg/kg valproate with radioactive tracer ( ^3^H-valproate). Dams of the chronic group were treated with a special feed containing valproate for at least two weeks prior to mating whereas dams of the acute group received a control feed. Non-pregnant female adult rats in the chronic group were treated with a special feed containing valproate for at least three weeks prior to experimentation. Each point represents the result from a single animal except for E19 thymus where a single sample was obtained by pooling (1 litter). Mean±SD. n=3–4.

Only limited data are available for E19 because the small size of the tissue required pooling to have enough to measure the labelled drugs. However, they indicate that, as with brain and CSF, entry of valproate appears to have been higher at E19 than at later ages. At P4, there was very little entry of ^3^H-valproate following injection of either 30 or 100 mg/kg. However, with 100 mg/kg chronic treatment or acute 200 mg/kg dose, there was a substantial and significant (p<0.05–0.01) increase compared with the lower doses. For lamotrigine, there appeared to be no significant differences in the entry for the different doses and the entry at each age was similar ( Figure 10). The thymus/plasma ratios for a single intraperitoneal injection of 100 mg/kg valproate or 20 mg/kg lamotrigine at E19, P4 and adult are shown in Figure 11. The entry of valproate at E19 may have been greater than later but only one pooled sample was available. There did not appear to be any age-related differences for lamotrigine. Combining doses of valproate and lamotrigine did not appear to have much effect on the entry of either drug ( Figure 12). There were no differences for valproate entry following acute and chronic treatment ( Figure 13).

### Measurement of valproate by LC–MS

**Figure 14.**
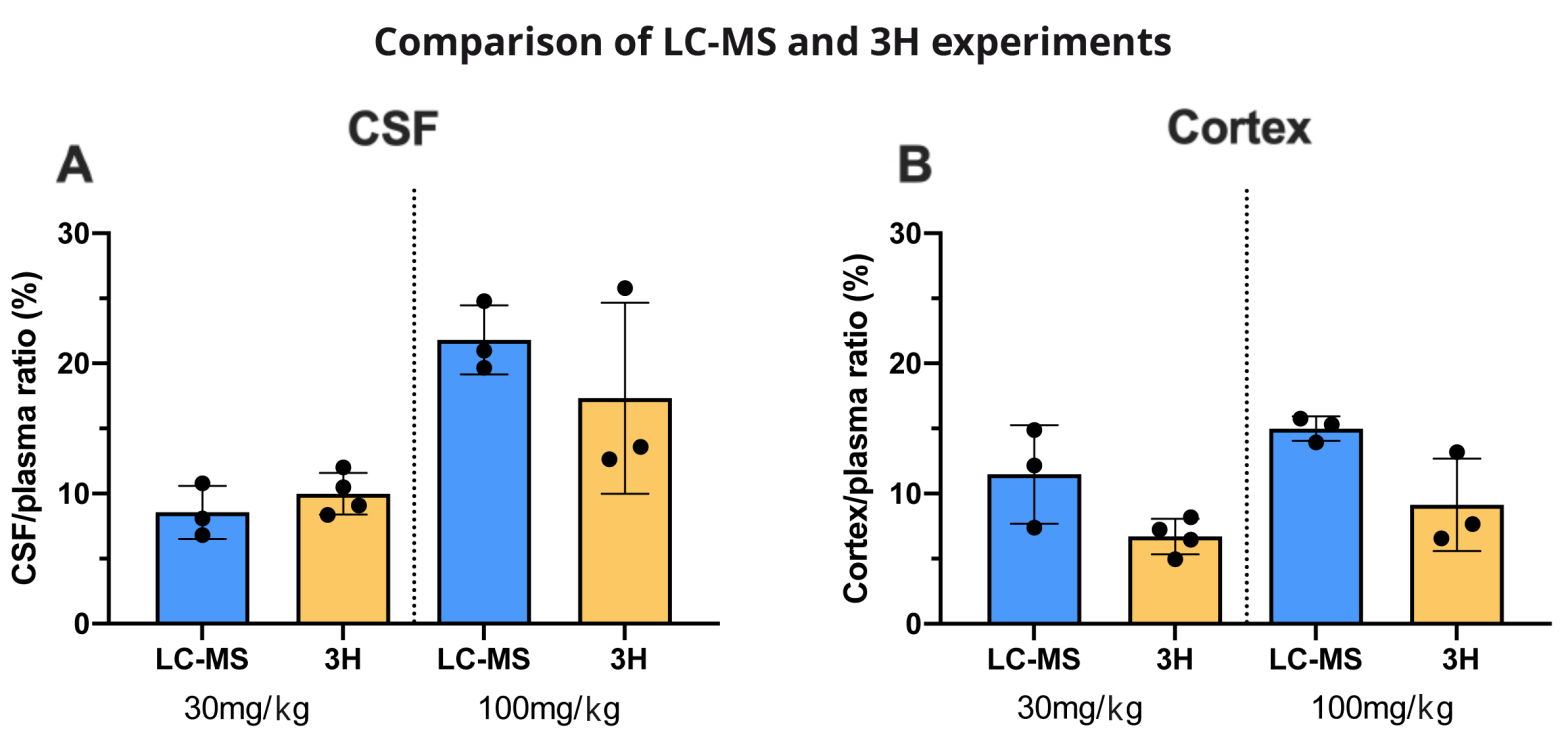
Valproate brain entry: comparison between LC/MS and ^3^H experiments. **A**. CSF/plasma and **B**. Cortex/plasma concentration ratios (%) of valproate in non-pregnant female adults, collected 30 min after *i.p*. injection of 30 mg/kg or 100 mg/kg valproate with or without radioactive tracer ( ^3^H-valproate). LC–MS, liquid chromatography–mass spectrometry. Each point represents the result from a single animal. Mean±SD. n=3–4.

Valproate concentrations were estimated by LC–MS in plasma, CSF and brain of adult and P4 rats ( Table 3). Following a single dose of 30 mg/kg (within the clinical range in adult patients, Johannessen & Johannessen, 2003) the plasma levels in adult rats were 34±8 µg/mL and in P4 pups 67 ± 12 µg/mL. At a dose of 100 mg/kg, a dose that is above the usual clinical range, the estimated plasma levels in experimental animals at both ages were also above the clinical range (141±6 µg/mL at P4 and 166±28 µg/mL in adults, Table 3). In order to compare and validate the two methods (LC–MS and radioactivity counting), CSF/plasma and brain/ plasma ratios were compared and were not significantly different at either age ( Figure 14).

**Table 3.** Concentration (μg/ml) of valproate in plasma, CSF and brain. P4 and non-pregnant female adult rats. collected 30 minutes after a single intraperitoneal injection of 30mg/kg or 100mg/Kg valproate; measured by LC–MS. Mean± SD. n=3–4. ^*^ Johannessen & Johannessen, (2003). Clinical dose range 20–60 mg/kg: *The Australian Medicines Handbook*, *US Food and Drug Administration (2016)*.

Injected dose	Concentration (μg/mL)		
	Plasma	CSF	Cortex
P4 30 mg/kg	67 ± 12	25 ± 1	17 ± 2
P4 100 mg/kg	141 ± 6	80 ± 6	79 ± 11
Adult 30 mg/kg	34± 8	3± 1	5± 1
Adult 100 mg/kg	166 ± 28	36 ± 3	29 ± 6
Clinical plasma range	40 – 100 *	-	-

## Discussion

This study provides data on the role of barriers in the brain and placenta in modulating entry of valproate and lamotrigine into the developing brain, when used alone or in combination. The rat has been used at an embryonic stage of development (E19), a postnatal age (P4) and adult. Drug entry into the CSF, cortex, brainstem has been estimated using radiolabelled drugs and compared with the thymus as this organ is known to have limited expression of one of the main efflux transporters, P-glycoprotein ( Valente *et al*., 2008) for which lamotrigine and some other anti-epileptic drugs are thought to be a substrate ( Ambroziak *et al*., 2010; Baltes *et al*., 2007; Jentink *et al*., 2010; Potschka *et al*., 2002; Rizzi *et al*., 2002).

The blood–brain, blood–CSF and placental barriers could be expected to contribute to the overall protection of the developing brain *in utero*; however, once born, the pup’s brain would rely solely on the protection provided by its own barriers. It has been previously reported that this protection is developmentally regulated for some drugs such as digoxin, cimetidine and paracetamol ( Koehn *et al*., 2019) but the degree of protection for valproate and lamotrigine is not known.

### Valproate concentrations achieved in rat plasma measured by LC–MS

Clinical doses of valproate range from 20–60 mg/kg ( Johannessen & Johannessen, 2003). We estimated valproate concentration in plasma, CSF and cortex of adult and P4 pups following an acute *i.p.* injection of either 30 mg/kg or 100 mg/kg. The dose of 30 mg/kg is well within the clinical range but 100 mg/kg is above. This was chosen on purpose to cover a wide range of drug doses as the route of administration we applied was different to that used in clinic ( *i.p. versus* oral). Nevertheless, following a single dose of 30 mg/kg, concentrations of valproate measured by LC–MS in plasma from P4 and adult rats were within (adult) or close to (P4) the clinical range ( Table 3). The higher dose of 100 mg/kg was above the recommended clinical concentration in patients’ plasma at both ages but clinical doses refer to valproate concentration at a steady level, not within 30 min of administration. The two methods (LC–MS and radioactivity counting) were also used for comparison to express results as ratios for CSF/plasma and brain/plasma in adults. These were very similar ( Figure 14).

### Dose-dependent drug entry

Entry of valproate into the CSF and brain was lower in the older animals studied ( Figure 3 and Figure 5). For a dose of 30 mg/kg (within the clinical range, Johannessen & Johannessen, 2003) and 100 mg/kg above the usual clinical range there was no difference in entry at any of the three ages studied. For the even higher dose of 200 mg/kg at P4 and adult, there was a small but significant (p<0.01–0.001) increase in entry of the drug. In the placenta, there was a substantial increase in fetal/maternal plasma ratio for the 100 mg/kg dose ( Figure 9B and further discussion below). These findings suggest that even above the clinical dose range brain barrier mechanisms were able to deal with the increased dose of valproate, in spite of the lesser effectiveness of the placenta. The mechanisms that might be involved are discussed below. The greater entry of the highest dose tested provides support for the current clinical practice of reducing valproate dose at the later stages of pregnancy, in order to limit the risks of cognitive impacts ( Thijs *et al*., 2019; Tomson *et al*., 2015; Tomson *et al*., 2019).

For lamotrigine at the doses studied, there was little difference in entry into CSF and brain at each dose ( Figure 4). But unlike valproate and other dugs examined in similar studies ( Koehn *et al.*, 2019) the level of lamotrigine entry appeared to increase in CSF and brain with age ( Figure 5). This suggests that the mechanisms protecting the brain against entry of lamotrigine are well established at E19 but appear less effective at older ages. Unlike valproate at the placental barrier, increasing the dose of lamotrigine did not affect the entry of the drug into the fetus ( Figure 9D) suggesting that the placental protective mechanisms were effective in this dose range (see below for further discussion).

Pregnancy registries and clinical studies have reported small numbers of a wide range of abnormalities in offspring of mothers that took lamotrigine, but mainly when exposure was in early pregnancy ( Briggs *et al.*, 2017). It is not clear whether the higher than adult rate of lamotrigine entry into brain at E19 represents a risk to the fetus and offspring when lamotrigine exposure is later in pregnancy.

### Effect of combining valproate and lamotrigine on entry of either drug

In experiments in which entry of 30 mg/kg ^3^H-valproate was measured in the absence or presence of different doses of lamotrigine ( Figure 6) or the converse of measuring entry of 6 mg/kg lamotrigine in the absence or presence of 30 mg/kg valproate ( Figure 7) there was little influence on drug entry by the combinations at the three ages studied. The dose combinations used were within that employed clinically (see Methods) which provides some reassurance that the current clinical practice of using lower doses of antiepileptic drugs in combination does not result in interference in brain entry between the drugs. This finding is thus relevant to the clinical practice of attempting to mitigate the ill effects of valproate by using lower doses combined with a second antiepileptic drug. Further studies could involve a wider range of doses and other antiepileptics with which valproate has been combined, particularly with longer term exposure to the drugs.

### Effect of chronic treatment on valproate entry

The chronic treatment used in the present study was that developed by Jazayeri *et al*. (2020) who showed that a feed containing 20 g/kg valproate resulted in blood levels in pregnant rats similar to those in pregnant mothers on treatment with this antiepileptic drug. At E19 there was a small but significant (p<0.05) decrease in cortical brain plasma ratios following injection of a dose of 100 mg/kg that included ^3^H-valproate compared with an acute injection ( Figure 8). At P4, there were no differences in the ratios for brain cortex, brainstem or CSF ( Figure 8). In adults, there was a significant decline (p<0.05) in ratio for both cortex and brainstem from about 10% to 6%, but no difference for CSF ( Figure 8). These reductions in entry at E19 and in adults are small and may not be functionally significant. However, the decline in brain ratios at E19 and in the adults may reflect an upregulation in the mechanisms that limit entry of valproate into the brain, as considered below. The lack of such an effect at P4 may just reflect the variation in response to valproate that sometimes occurs in patients. 

### Placental permeability

Fetal/maternal plasma ratios were used as an estimate of drug entry into the fetus and as an indication of the level of protection provided by the placenta. For valproate, in acute dose experiments, there was a significant increase from 38.2±4.4% with a dose of 30 mg/kg to 71.3±11.5% following a dose of 100 mg/kg ( Figure 9B, p<0.0001). This suggests the possibility of a mechanism limiting entry of valproate across the placenta that can be saturated at higher doses. Following chronic treatment the fetal/maternal plasma ratio was reduced to the same as 30 mg/kg valproate, indicating the possibility of upregulation of efflux mechanisms. It is of potential clinical significance that entry into the fetus with chronic valproate treatment was reduced substantially ( Figure 9B) with a much smaller effect on the clinically important entry of valproate into the mother’s brain ( Figure 8).

For lamotrigine there was no significant difference in the fetal/maternal plasma ratios for the two doses tested ( Figure 9D).

### Thymus permeability

Thymus tissue was collected in this study because it has been found to contain limited expression of the ABC efflux transporter P-glycoprotein (Abcb1) a much-studied efflux mechanism that excludes or limits the entry of many drugs at brain barriers; there is some evidence that lamotrigine may be a substrate ( Zhang *et al*., 2012 and see below). The residual vascular space was much larger in the thymus than in the brain regions sampled Figure 1) indicating a much larger vascularization of the tissue. This means that corrections applied for drug in blood in the tissue samples collected were much larger in the case of the thymus. At E19, the data for thymus are limited because measurement required pooling of tissue. However, the level of entry of valproate and lamotrigine appeared to be similar in thymus and brain and was not affected by increases in the drug doses ( *c.f.* Figure 3, Figure 4, Figure 10).

At P4 for doses of 30 and 100 mg/kg the entry was substantially less than at E19 or in the adult but increased with a dose of 200 mg/kg valproate. The thymus thus appears to have effective mechanisms limiting entry of valproate in the postnatal period, a time when the development of the thymus has important biological functions ( Miller, 2020). Entry of lamotrigine was not much different at the different ages suggesting that efflux mechanisms for the drug develop early.

Addition of lamotrigine to valproate did not affect the entry of valproate into the thymus ( Figure 12) whereas addition of valproate to lamotrigine increased the entry of lamotrigine from a ratio of 191.9±23.2% to 286.3±36.8% (p<0.05) at P4 but not in adults ( Figure 12). Chronic treatment with valproate did not appear to affect its entry into the thymus ( Figure 13).

### Mechanisms limiting entry of valproate and lamotrigine across the placenta and into brain and CSF

The main mechanisms that limit drug entry into the brain are the ABC efflux transporters and a few SLC transporters that are bidirectional ( Han *et al*., 2018; Roberts *et al*., 2008; Saidijam *et al*., 2018; Strazielle & Ghersi-Egea, 2015). In the case of valproate, the evidence is conflicting. This is partly due to a substantial use of *in vitro* systems some of which give different results depending on the cells used ( Grewal *et al*., 2017; Zhang *et al*., 2012) or the species from which the cells were derived ( Alms *et al*., 2014). *In vivo* studies may suffer from the limitation that generally effects of valproate (and other antiepileptics) on ABC transporter gene expression or changes in protein functionality were carried out using samples of whole brain. This does not allow distinguishing between effects on ABC transporters in cerebral endothelial cells from brain parenchymal cells. In a study in rabbits Scism *et al*. (2000) used probenecid, an organic anion transporter inhibitor and micro dialysis to measure drug concentrations in brain extracellular fluid of valproate administered intravenously and compared that with estimates of plasma and brain levels of valproate. Their measurements showed that probenecid increased intracellular brain tissue valproate 2.5 times but did not affect extracellular fluid/plasma concentration ratios taken as an estimate of blood–brain barrier transfer. Thus an additional factor to be taken account of in studies of barrier mechanism in relation to drug entry is the localisation of potential ABC or other efflux transporters. Wang *et al*. (2013) avoided this problem by studying the transport of a P-glycoprotein substrate in freshly isolated mouse cerebral capillaries. They reported that valproate increased the transfer of the specific P-glycoprotein fluorescent substrate NBD-CSA, suggesting that valproate itself interacts with P-glycoprotein. Weiss *et al.* (2003) have also shown that valproate, lamotrigine and verapamil increased entry of the fluorescent P-glycoprotein substrate calcein-AM into LLC-PK1 cells transfected with human MDR1 and into primary cultures of porcine brain capillary endothelial cells. However, this was only seen at high valproate and lamotrigine concentrations and to a much lower level (+10%) compared with the high-affinity P-glycoprotein substrate verapamil. In contrast, Moerman *et al*. (2011) found that *in vivo* in mice valproate did not interact with P-glycoprotein at therapeutic or higher doses. In support of this finding is the report that P-glycoprotein deficient Mdr1a/b(-/-) mice in the intrahippocampal kainate model of mesial temporal lobe epilepsy there we no significant differences in the anti-seizure efficacy of valproate and lamotrigine ( Bankstahl *et al*., 2016).

In a comprehensive study using both *in vitro* and *in vivo* methods, Baltes *et al*. (2007) concluded that there is no evidence that valproate is a substrate for P-glycoprotein.

Thus on balance current evidence suggests that valproate is unlikely to be a P-glycoprotein substrate at the brain barriers.

*In vitro* studies using MDCKII cells transfected with human BCRP or mouse Bcrp did not find any evidence of valproate transport by BCRP ( Cerveny *et al*., 2006; Römermann *et al*., 2015). 

It is not clear what mechanism(s) limit entry of valproate across the placental barrier. Jinno *et al*. (2020) carried out a detailed study of 8 ATP and 10 SLC transporters at E13 and E20 using RTqPCR in pregnant rats. Abcb1a, 1b, Abcc2, Abcc4 were upregulated between these ages as were Slc7a5, Slc16a3, Slc22a3, Slc22a4, Slco2b1, Slco4a1. Effects of a single dose and treatment over 4 days with valproate were also studied. In response to multiple doses of valproate Mdr1a (P-glycoprotein Abab1a) and Mrp4 (Abcc4) were increased over 2-fold at E20, which may account for the decreased entry of valproate across the placenta observed in our experiments ( Figure 9) in response to chronic treatment.

In the case of lamotrigine, there have been some conflicting reports, but the accumulating evidence from *in vitro* and *in vivo* studies is that lamotrigine is a low affinity substrate for both BCRP and P-glycoprotein. Zhang *et al*. (2012) summarize evidence from patient data, *in vivo* and *in vitro* experiments supporting this with additional recent human data available from Domjanović *et al*. (2018). In addition there has been a report of active transport of lamotrigine into brain via the OCT1 transporter ( Dickens *et al*., 2012). 

Potschka *et al*. (2002) provided evidence that lamotrigine was a substrate for P-glycoprotein *in vivo* with increased entry of lamotrigine into rat brain extracellular fluid when a competing P-glycoprotein substrate (verapamil) was present. These authors used twin dialysis probes with guide catheters, implanting one probe into the left frontal cortex and the other into the right. Lamotrigine was administered to the animals *i.p.* and then verapamil infused *via* one of the guide catheters to one frontal cortex whilst the other side acted as a vehicle only control. The concentration of lamotrigine in the dialysate from the verapamil infused cortex was found to be approximately double that measured in the probe from the control hemisphere in the same animals.

### Potential clinical relevance of the findings from present experiments in rats

For valproate, tissue/plasma ratios in E19 fetuses were not significantly affected by a large difference in doses used in the study (30 mg/kg compared to 100 mg/kg, Figure 3). However, the ratios were higher than at P4 and much higher than in the adult for the same doses ( Figure 3 and Figure 5). Placental transfer at E19 considerably increased for 100 mg/kg dose compared to 30 mg/kg dose ( Figure 9B). The lesser protection against the higher dose combined with the much higher tissue/plasma ratios suggests that fetal brain may be particularly susceptible to effects of valproate administered in late-gestation rats; a comparable stage in human brain development would be the first trimester ( Clancy *et al*., 2013). At both P4 and in adults the CSF/plasma and brain/plasma ratios increased with increasing doses of between 10 mg/kg and 200 mg/kg ( Figure 3). The ratios were approximately two-fold higher at P4 than in the adult. The progressive decline in ratios between E19 and adult suggest the development of brain barrier protective mechanisms against valproate. The nature of such mechanisms is unclear (see previous section).

The CSF/plasma and brain/plasma ratios for lamotrigine were similar at P4 and in adults and were not affected by increasing the dose ( Figure 4). At E19 the ratios were significantly lower for a dose of 20 mg/kg in the CSF and cortex ( Figure 4). This suggests that brain barrier protective mechanisms against lamotrigine are developed as early as E19 (equivalent to 13–14 weeks gestation in human, Clancy *et al*., 2013) but appear less effective at older ages ( Figure 5). Current evidence suggests that the mechanisms involved could include the ABC efflux transporters P-glycoprotein and BCRP (see previous section).

Adding 6 mg/kg lamotrigine to 30 mg/kg valproate (doses that are similar to the clinically used range, see Methods) at E19 and adult and up to 20 mg/kg at P4 ( Figure 6) did not affect the CSF/plasma, brain/plasma or fetal/maternal plasma ratios of valproate.

This is an important validation of the current clinical approach to control seizures during pregnancy by reducing valproate doses by adding lamotrigine ( Tomson *et al*., 2019). The increase in CSF/plasma ratio of valproate in the presence of 6 mg/kg lamotrigine in the adult and much smaller non-significant increase in the brain are not relevant to the antiepileptic drug status of the developing brain, but might indicate that the clinical dose of valproate for treatment of the mother could be reduced further.

In the converse experiment of adding 30 mg/kg valproate to 6 mg/kg lamotrigine in P4 and adult rats the ratios were not affected by the addition of valproate except for a small increase in the cortex but not brainstem at P4 ( Figure 7); this further supports the clinical use of this drug combination in pregnant patients with epilepsy.

Results from experiments in which female rats were fed a diet containing 20 g/kg valproate from before pairing and throughout pregnancy were compared with results from female rats fed on chow without the drug. At E19 there was a small but significant reduction in cortex/plasma ratio ( Figure 8) and a substantial significant reduction in the fetal/maternal plasma ratio in the chronically treated animals from 70% to 35% ( Figure 9B). For patients delivered at term this ratio was reported to be 120% ( Bank *et al*., 2017). The difference might be due to the difference in gestation at which the measurements were made or reflect greater protection by the rat placenta.

At P4, the various ratios were unaffected by chronic treatment but in the adults the cortex and brainstem ratios were reduced in the chronically treated animals ( Figure 8). These reductions in some ratios in the treated animals suggest that there may be a degree of upregulation of the mechanisms limiting valproate entry across barrier interfaces, as described previously for other drugs ( Koehn *et al*., 2019 and see previous section). The relatively large decrease in fetal/maternal plasma ratio for valproate in chronically treated pregnant animals is of clinical significance as it suggests an increased degree of fetal protection with only a smaller effect on valproate entry in the maternal brain, which is important for treatment of the mother. Löscher (2007) has provided a valuable comparison in rats and humans of the pharmacokinetics of antiepileptic drugs including valproate and lamotrigine. The results from LC–MS measurement of valproate gave values in plasma in adult rats that were within the clinical range for a dose of 30 mg/kg, a dose that is with the range used in patients. This confirms that doses to be used can be based on body weight for such animal experiments. For this dose at P4, the plasma level was above the normal clinical upper limit ( Table 3); this is hardly surprising given likely differences in drug metabolism and plasma protein binding in immature animals. It does suggest that in future experiments in developing animals it may be appropriate to scale down the amount administered.

### Limitations of study

This study was carried out during two periods of lockdown in Melbourne due to the Covid-19 pandemic. In the first period from Easter to mid-June, our laboratories were closed and the animal house ran down its breeding colonies because of uncertainty about how long the lockdown would continue. We were allowed to re-open our laboratories in mid-June under considerable restrictions on the number of people allowed in the lab and the periods they could be there for. These restrictions continued throughout the lockdown (late July to early November) and were still current into 2021. The staff restrictions and limited supply of pregnant and postnatal animals made it difficult to plan adequate numbers of experiments. However, it has been possible to carry out a comprehensive range of experiments on valproate and lamotrigine alone and in combination as well as some limited chronic experiments using valproate-containing feed. The results are internally consistent and show clear developmentally related patterns and no results have previously been published in which drug levels have been examined in fetal and postnatal blood and CSF. Such measurements are essential for determining the relative quantitative roles of the placental, blood-brain and blood-CSF barrier interfaces for antiepileptic drugs. It was only possible to study one dose of one of the drugs (valproate) in chronic experiments in which valproate was incorporated into the feed. It took several months to obtain the feed and the experiments took 5–6 weeks to conduct and only when suitable animals were available. However, this feeding regime has been shown previously to achieve clinically appropriate blood levels of the drug ( Jazayeri *et al*., 2020). We have used radiolabelled drugs for most of the studies as liquid scintillation is a highly sensitive method that was able to detect measurable amounts of labelled drug in the limited volumes of fetal CSF and plasma available. However, these results have been validated by some estimations of valproate using LC–MS.

It is, of course, important to be cautious about extrapolating results from animal studies to humans. However, we are dealing with fundamental biological mechanisms so that, although there may be differences in detail, it is reasonably likely that the overall pattern of results will be significant for the human condition. There is a wealth of information which allows brain development to be compared in many species but particularly humans and rodents ( Clancy *et al*., 2013; Workman *et al*., 2013). Also, the placentas of these species are classified as haemochorial and although there are some structural differences they are much more similar than for example the much “tighter” epitheliochorial multicotyledonary placenta of the sheep ( Schneider, 1991) a species that has been used for many valuable developmental studies but are born at an advanced stage of brain development compared with humans ( Clancy *et al.*, 2013).

## Conclusions and significance of the study

Studies described here show that in the late gestational rat the placenta provides a significant impediment to transfer of the antiepileptic drugs valproate and lamotrigine, from the mother to the fetus but the degree of protection was less at higher doses of the drugs. In chronic experiments with valproate delivered *via* the feed there was evidence of a substantial upregulation in placental protection after a prolonged exposure to the drug. At the blood–brain and blood–CSF barriers, the results show that entry of the drugs at E19 is high but declines with age, indicating the development of protective mechanisms at these interfaces. In experiments using different combinations of doses of valproate and lamotrigine there was generally no difference in drug entry when compared to entry of either drug alone. This provides support for the current clinical practice of using lamotrigine to reduce the amount of valproate required to control seizure where valproate in some patients is the only effective antiepileptic drug; this limits the potential for this drug to cause congenital malformations. The finding of higher entry of valproate and possibly other AEDs still to be investigated early in brain development. The decline as the brain matures, provides a basis for future studies of the mechanisms involved and for studies of potential deleterious effects on brain development and behaviour in the offspring of pregnant females treated with AEDs.

## Data availability

### Underlying data

Figshare: Underlying data for ‘Entry of antiepileptic drugs (valproate and lamotrigine) into the developing rat brain’. https://doi.org/10.26188/14374379 ( Toll *et al.*, 2021)

The project contains the following underlying data:

- Raw data for liquid scintillation experiments:
  • Lamotrigine adult: CPM, DPM and weight for all samples in adult lamotrigine experiments
  • Lamotrigine P4: As above
  • Lamotrigine E19: As above
  • Valproate adult : As above
  • Valproate P4: As above
  • Valproate E19: As above
- Raw data for LC-MS:
  • 210319: Raw data files for measurements on 19 ^th^ March 2021
  • 210330: Raw data files for measurements on 30 ^th^ March 2021
  • 210402: Raw data files for measurements on 2 ^nd^ April 2021
  • LCMS peakarea: Peak area & concentration calculation for all samples
  • LCMS Skyline template: Template used to extract peak area from raw data in Skyline

### Extended data

Figshare: Extended data for ‘Entry of antiepileptic drugs (valproate and lamotrigine) into the developing rat brain’. https://doi.org/10.26188/14374379 ( Toll *et al.*, 2021)

This project contains the following extended data:

•   Tables of numerical values for each figure

Data are available under the terms of the Creative Commons Attribution 4.0 International license (CC-BY 4.0).
