# Supplementary material for: Entry of antiepileptic drugs (valproate and lamotrigine) into the developing rat brain
Source: F1000Res. 2021 Jul 26;10:384. Originally published 2021 May 13. [Version 2] doi: 10.12688/f1000research.52607.2 (PMC8207807; doi:10.12688/f1000research.52607.2)
Supplement: Peer review history

# Peer review: Reviewer response for version 1

**DOI:** 10.5256/f1000research.55908.r86365

**Published:** 2021-06-24

**Authors:** Stefan Liebner, Elif Fidan

**Affiliations:** 1 Institute of Neurology (Edinger-Institute), Interdisciplinary Centre for Neuroscience Frankfurt, Frankfurt, Germany

Toll *et al*. have evaluated in the present manuscript the entry of anti-epileptic drugs into the developing rat brain. Specifically, they have explored the entry of valproate, known as the most effective treatment for maternal epilepsy, together with the accessory drug lamotrigine. Considering the fact that valproate is mostly combined with a second anti-epileptic drug since its high doses lead to congenital abnormalities, Toll *et al.* have studied the entry of the drugs valproate and lamotrigine when applied alone and in combination. Sprague-Dawley rats were used at three stages, including embryonic day (E)19, pups at postnatal (P) day 4, and adults since these ages represent developmental milestones that can be translated to stages of human brain development, corresponding to the end often first trimester, late second trimester and adult. Moreover, the authors compared acute versus chronic treatment of pregnant rat with valproate. The entry of valproate into brain and CSF was identified to be higher at E19 and P4 compared to adult stage. The entry into the brain was not observed to be dose-dependent, whereas the transfer of valproate across the placenta increased with its dose and was highest at 100mg/kg.

 Instead, lamotrigine did not show significant differences regarding its permeability between the three stages. Nor did the combination treatment of valproate with lamotrigine show significant differences in brain entry of the drugs. Chronic treatment of pregnant rats with valproate resulted in reduced brain entry of valproate as well as in decreased placental transfer.

 The presented data in this manuscript are highly relevant and interesting to the field of clinical epilepsy research. The work may have impact on the treatment of pregnant women suffering from epilepsy.

 However, to augment the scientific merit of the manuscript, some issues require the author's attention:

**Major points:**

 The major points of critique on the manuscript by Toll *et al.* are listed below:

- Figure 3E shows that there is a significant difference in the entry of 30 and 100 mg/kg valproate into cortex at P4. However, the authors claim on Page 13, in Paragraph 2: "For a dose of 30 mg/kg (within the clinical range, Johannessen & Johannessen, 2003) and 100 mg/kg above the usual clinical range there was no difference in entry at any of the three ages studied." The authors should comment on this discrepancy.
- Figure 1 shows the residual vascular space in the organs cortex, brainstem, and thymus. The authors report that each point represents the result from a single animal. Assuming that one animal was used to collect these three organs, why is the number of animals different for the different organs? For example the figure shows in A that n=3 animals were used to analyze the residual vascular space in the adult cortex, while the number of animals for the analysis of the brainstem is only n=2.
- There is a high variation in the n numbers between different groups and/or different time points. Moreover, in several cases, the n number is not high enough to perform statistical analysis. For instance, in Figure 4E, one of the three animals might be outlier, suggesting that more replicates are needed. The authors should comment on this.
- The authors have evaluated the entry of ^3^H-valproate into the cortex of E19 fetuses following chronic feeding of the mother. Considering the idea of combining valproate with lamotrigine, additional studies in which the entry of the drugs when combined in a chronic treatment paradigm would be complementary.
- It is not very clear in the methods part whether the animals in the chronic group received 100 mg/kg i.p. valproate injection or not. If they did, isn’t it a combination of chronic+acute instead of just a chronic phase? If they did not, isn’t this time frame following the i.p. injection too short to evaluate a chronic phase outcome?
- As it is written in the figure legend and on Page 12, Paragraph 6, Figures 9A and 9C show results from only one animal for each time point which does not seem to be sufficient to draw a conclusion. Moreover, Figures 9B and 9C show aggregated data along the time frame following the injection. A setup in which enough number of replicates were used for each time point and in which the aggregated data is from those replicates for each time point would be better to understand the dose and time-dependent entry of the drug into the fetus.
- The authors write in the thymus permeability section: "At P4 for doses of 30 and 100 mg/kg the entry was substantially less than at E19 or in the adult but increased with a dose of 200 mg/kg valproate." However, Figures 10B and C show that valproate entry to the thymus at 100 mg/kg dose is higher in P4 compared to the adult. The authors should clarify this.
- Considering the increase in the entry of lamotrigine to CSF and brain with age, it is suggested in the manuscript that mechanisms protecting the brain against entry of lamotrigine are well established at E19 but appear less effective at older ages. This sounds like an assertive explanation for the observed phenomena with statistically not satisfying numbers of replicates. Given that lamotrigine potentially is a substrate of P-glycoprotein, the authors should comment on the regulation of the ABC transporter between E19 and postnatal stages up to adulthood.
- The "Limitations of the study" part does not add anything to the scientific quality of the article. These limitations might be valid for any research group during the pandemic. The reviewer can only judge if the presented data are valid and if experimental and statistical rigidity were applied. Why and how it came about, and their ability to present solid data, cannot be judged by the reviewer.

**Minor points:**

- Page 1, Results section: The sentence "Entry of valproate into brain and CSF was higher at E19 and P4 compared to adult but was not dose-dependent;.." could be written in a different way to make it clear that valproate entry is dose dependent but only at E19 this is not the case.
- Page 3, line 69: Check the format of the reference "(rat, Koehn *et al*., 2019; Koehn *et al*., 2020)". Was the word "rat" supposed to be in the reference?
- Page 7: "The ratio of 3H-dextran into cortex and brainstem compared with plasma, illustrated in Figure 1A and B, showed no significant difference at any of the ages studied and was around 2–4%." Was it supposed to be "entry into" instead of just "into"?
- Figure 9C: Why the 20mg/kg case lasts only until app. 95 min? It is written on Page  12 "Figures 9B and 9D show the aggregated data up to 110 min." However, the 20mg/kg does not reach that time point.
- Figure 10: Why is "Thymus" written on top of some graphs while on top of others not? This "subtitle" is not needed anyway, since there is already main title of the figure.

Overall the manuscript by Toll *et al.* provides interesting and novel data on the treatment of pregnant rats with the anti-epileptic drugs valproate and lamotrigine.

 Nevertheless, in its present form, the manuscript requires some additional work to augment its scientific merit. Hence the reviewer recommends the manuscript for indexing after major revision.

Is the work clearly and accurately presented and does it cite the current literature?

Yes

If applicable, is the statistical analysis and its interpretation appropriate?

Partly

Are all the source data underlying the results available to ensure full reproducibility?

Yes

Is the study design appropriate and is the work technically sound?

Partly

Are the conclusions drawn adequately supported by the results?

Partly

Are sufficient details of methods and analysis provided to allow replication by others?

Yes

Reviewer Expertise:

Vascular biology, blood-brain barrier research, brain and vascular development, cell-cell communication and signaling

We confirm that we have read this submission and believe that we have an appropriate level of expertise to confirm that it is of an acceptable scientific standard, however we have significant reservations, as outlined above.

# Response

**Published:** 2021-07-18

**Authors:** Norman Saunders

**Affiliations:** Department of Pharmacology and Therapeutics, University of Melbourne, Australia

We thank Drs Liebner and Fidan for their detailed appraisal of our paper. We list our responses below. Where appropriate changes in the ms have been tracked.

**Major points:**

 The major points of critique on the manuscript by Toll *et al.* are listed below: 

- Figure 3E shows that there is a significant difference in the entry of 30 and 100 mg/kg valproate into cortex at P4. However, the authors claim on Page 13, in Paragraph 2: "For a dose of 30 mg/kg (within the clinical range, Johannessen & Johannessen, 2003) and 100 mg/kg above the usual clinical range there was no difference in entry at any of the three ages studied." The authors should comment on this discrepancy.

*We thank the reviewers for drawing out attention to this discrepancy which has now been corrected.*

*“For a dose of 30 mg/kg (within the clinical range, Johannessen & Johannessen, 2003) and 100 mg/kg (above the usual clinical range) there was no difference in entry into CSF at any of the three ages studied; in cortex and brainstem the entry of valproate was higher at P4 with the larger clinical dose, but not at the other ages (Figure 3).”*

  

- Figure 1 shows the residual vascular space in the organs, cortex, brainstem, and thymus. The authors report that each point represents the result from a single animal. Assuming that one animal was used to collect these three organs, why is the number of animals different for the different organs? For example the figure shows in A that n=3 animals were used to analyze the residual vascular space in the adult cortex, while the number of animals for the analysis of the brainstem is only n=2.

*We initially only took cortex but later on decided to investigate brainstem as well so the first adult used for dextran blood space didn’t have a brainstem value.*

  

- There is a high variation in the n numbers between different groups and/or different time points. Moreover, in several cases, the n number is not high enough to perform statistical analysis. For instance, in Figure 4E, one of the three animals might be outlier, suggesting that more replicates are needed. The authors should comment on this.

*We do comment on this in “Limitations of the Methods” and we agree, that more experiments would have been desirable.*

  

- The authors have evaluated the entry of ^3^H-valproate into the cortex of E19 fetuses following chronic feeding of the mother. Considering the idea of combining valproate with lamotrigine, additional studies in which the entry of the drugs when combined in a chronic treatment paradigm would be complementary.

*We agree with this point and these experiments are planned.  It takes considerable time to prepare feeds, check if animals consume enough to obtain a clinical range of the drug in plasma and make sure they are willing to mate when on this treatment.*

  

- It is not very clear in the methods part whether the animals in the chronic group received 100 mg/kg i.p. valproate injection or not. If they did, isn’t it a combination of chronic+acute instead of just a chronic phase? If they did not, isn’t this time frame following the i.p. injection too short to evaluate a chronic phase outcome?

*All animals with chronic feeding received a final injection with radiolabelled valproate (described on page 6). The final acute injection is used to measure permeability to find out whether chronic exposure alters this aspect of the barrier mechanisms and therefore drug entry (see Koehn et al, 2019).*

- As it is written in the figure legend and on Page 12, Paragraph 6, Figures 9A and 9C show results from only one animal for each time point which does not seem to be sufficient to draw a conclusion. Moreover, Figures 9B and 9C show aggregated data along the time frame following the injection. A setup in which enough number of replicates were used for each time point and in which the aggregated data is from those replicates for each time point would be better to understand the dose and time-dependent entry of the drug into the fetus.

*Each point is one fetus but the whole line graph shows data for that litter. Fetuses are taken one after the next therefore there will always be a time lag between littermates.*

  

- The authors write in the thymus permeability section: "At P4 for doses of 30 and 100 mg/kg the entry was substantially less than at E19 or in the adult but increased with a dose of 200 mg/kg valproate." However, Figures 10B and C show that valproate entry to the thymus at 100 mg/kg dose is higher in P4 compared to the adult. The authors should clarify this.

*We thank the reviewers for drawing our attention to this error which has been corrected.*

*“At P4 for the dose of 30 mg/kg the entry was substantially less than at E19 or in the adult but increased with a dose of 200 mg/kg valproate. At 100 mg/kg the entry declined with age.”*

  

- Considering the increase in the entry of lamotrigine to CSF and brain with age, it is suggested in the manuscript that mechanisms protecting the brain against entry of lamotrigine are well established at E19 but appear less effective at older ages. This sounds like an assertive explanation for the observed phenomena with statistically not satisfying numbers of replicates. Given that lamotrigine potentially is a substrate of P-glycoprotein, the authors should comment on the regulation of the ABC transporter between E19 and postnatal stages up to adulthood.

*As the reviewers comment lamotrigine is at best a potential substrate for P-glycoprotein. We have modified the statement in the text.*

*“One possible explanation for the observed age-related differences in the entry of LTG and VPA into the brain could be the time frame of when responsible transporters become functional (see Discussion in Koehn et al, 2019).”*

  

- The "Limitations of the study" part does not add anything to the scientific quality of the article. These limitations might be valid for any research group during the pandemic. The reviewer can only judge if the presented data are valid and if experimental and statistical rigidity were applied. Why and how it came about, and their ability to present solid data, cannot be judged by the reviewer.

*We disagree and note that two other Reviewers have not commented upon this aspect of the paper. We have nevertheless restricted the comment on the impact of the pandemic to a single sentence.  We feel strongly that all papers should carry a section detailing limitations. If more authors were willing to acknowledge the experimental limitations of their studies it might lead to an improvement in standards of research and help readers assess validity of results (e.g. Begley & Ioannidis, 2015, Circ Res. 2015;116:116-126. DOI: 10.1161/CIRCRESAHA.114.303819.).*

**Minor points:**

- Page 1, Results section: The sentence "Entry of valproate into brain and CSF was higher at E19 and P4 compared to adult but was not dose-dependent;.." could be written in a different way to make it clear that valproate entry is dose dependent but only at E19 this is not the case.

*Revised to read “Entry of valproate into brain and CSF was higher at E19 and P4 compared to adult and was dose-dependent, except at E19."*

  

- Page 3, line 69: Check the format of the reference "(rat, Koehn *et al*., 2019; Koehn *et al*., 2020)". Was the word "rat" supposed to be in the reference? Can remove?

*This was to indicate that the animal model was rat. Text modified to indicate this.*

  

- Page 7: "The ratio of 3H-dextran into cortex and brainstem compared with plasma, illustrated in Figure 1A and B, showed no significant difference at any of the ages studied and was around 2–4%." Was it supposed to be "entry into" instead of just "into"?

*Detectable dextran does not enter the brain in 5 minutes. The wording has been changed to “in brain tissue” to clarify this.*

  

- Figure 9C: Why the 20mg/kg case lasts only until app. 95 min? It is written on Page  12 "Figures 9B and 9D show the aggregated data up to 110 min." However, the 20mg/kg does not reach that time point.

*Usually fetuses are collected at 10 minutes intervals, the particular dam used for the 20mg/kg experiment had 5 pups. We limit the time to present results from all experiments to 110 min to allow longer experiments (more fetuses) to be comparable with shorter experiments.*

  

- Figure 10: Why is "Thymus" written on top of some graphs while on top of others not? This "subtitle" is not needed anyway, since there is already main title of the figure.

*Thank you for drawing our attention to this anomaly. We have asked for “Thymus” to be removed from Figure 10 B and C and Figure 12 B.*

# Peer review: Reviewer response for version 1

**DOI:** 10.5256/f1000research.55908.r86533

**Published:** 2021-06-15

**Authors:** Neil Dani

**Affiliations:** 1 Department of Pathology, Boston Children's Hospital, Boston, MA, USA

The authors evaluate antiepileptic drug entry into the developing brain in fetal rat models. They test entry of valproate and lamotrigine into the fetus by LC-MS and report higher valproate entry in the developing brain. Chronic valproate exposure was found to restrict entry into the fetal brain. Further, lamotrigine showed milder differences with age and dose dependence. Finally, no significant interactions were seen in the combination therapy models. Overall, the study is informative and provides dosing guidelines for future experiments that investigate anti-epileptic drug effects on fetal brain development.

Is the work clearly and accurately presented and does it cite the current literature?

Yes

If applicable, is the statistical analysis and its interpretation appropriate?

Yes

Are all the source data underlying the results available to ensure full reproducibility?

Yes

Is the study design appropriate and is the work technically sound?

Yes

Are the conclusions drawn adequately supported by the results?

Yes

Are sufficient details of methods and analysis provided to allow replication by others?

Yes

Reviewer Expertise:

Developmental neuroscience, neurobiology, blood-CSF barrier.

I confirm that I have read this submission and believe that I have an appropriate level of expertise to confirm that it is of an acceptable scientific standard.

# Response

**Published:** 2021-06-28

**Authors:** Norman Saunders

**Affiliations:** Department of Pharmacology and Therapeutics, University of Melbourne, Australia

We should like to thank Dr Dani for his review of our paper and his comment that our study will provide guidelines for future experiments. In fact, we are already embarking on these.

# Peer review: Reviewer response for version 1

**DOI:** 10.5256/f1000research.55908.r85603

**Published:** 2021-06-07

**Authors:** Alan Leviton

**Affiliations:** 1 Department of Neurology, Boston Children's Hospital's Hospital, Harvard Medical School, Boston, MA, USA

This study of rats was designed to evaluate the entry of two antiepileptic drugs' (valproate and lamotrigine) entry into developing brain. The antiepileptic drugs were injected into the peritoneal cavity of pregnant rats and the offspring were assessed at embryonic day (E) 19, postnatal day (P) 4, and when adults. Based on their findings, the authors conclude that “the placenta provides a significant impediment to transfer of the antiepileptic drugs valproate and lamotrigine from the mother to the fetus.”

 This is a thorough evaluation of the very drugs the fetuses of epileptic women are likely to be exposed to, and provides clinicians with the confidence they need to assure their patients that their anti-epileptic drugs are unlikely to damage their baby's brain.

Is the work clearly and accurately presented and does it cite the current literature?

Yes

If applicable, is the statistical analysis and its interpretation appropriate?

Yes

Are all the source data underlying the results available to ensure full reproducibility?

Yes

Is the study design appropriate and is the work technically sound?

Yes

Are the conclusions drawn adequately supported by the results?

Yes

Are sufficient details of methods and analysis provided to allow replication by others?

Yes

Reviewer Expertise:

neonatal neurology

I confirm that I have read this submission and believe that I have an appropriate level of expertise to confirm that it is of an acceptable scientific standard.

# Response

**Published:** 2021-06-28

**Authors:** Norman Saunders

**Affiliations:** Department of Pharmacology and Therapeutics, University of Melbourne, Australia

We should like to thank Dr Leviton for his review of our paper. We appreciate his comment that the study was a thorough evaluation and likely to be of value to clinicians in advising their patients who are required to take antiepileptic drugs during pregnancy.
